# Mg^2+^ Effect on Argonaute and RNA Duplex by Molecular Dynamics and Bioinformatics Implications

**DOI:** 10.1371/journal.pone.0109745

**Published:** 2014-10-17

**Authors:** Seungyoon Nam, Hyojung Ryu, Won-joon Son, Yon Hui Kim, Kyung Tae Kim, Curt Balch, Kenneth P. Nephew, Jinhyuk Lee

**Affiliations:** 1 Cancer Genomics Branch, National Cancer Center, Goyang, Kyunggi-do, Korea; 2 Korean Bioinformation Center (KOBIC), Korea Research Institute of Bioscience and Biotechnology (KRIBB), Daejeon, Korea; 3 Department of Bioinformatics, University of Sciences and Technology, Daejeon, Korea; 4 Department of Chemistry, North Carolina State University, Raleigh, North Carolina, United States of America; 5 Next Therapeutics Branch, National Cancer Center, Goyang, Kyunggi-do, Korea; 6 Molecular Epidemiology Branch, National Cancer Center, Goyang, Kyunggi-do, Korea; 7 Bioscience Advising, Indianapolis, Indiana, United States of America; 8 Medical Science Program, Indiana University School of Medicine, Bloomington, Indiana, United States of America; Wuhan University, China

## Abstract

RNA interference (RNAi), mediated by small non-coding RNAs (e.g., miRNAs, siRNAs), influences diverse cellular functions. Highly complementary miRNA-target RNA (or siRNA-target RNA) duplexes are recognized by an Argonaute family protein (Ago2), and recent observations indicate that the concentration of Mg^2+^ ions influences miRNA targeting of specific mRNAs, thereby modulating miRNA-mRNA networks. In the present report, we studied the thermodynamic effects of differential [Mg^2+^] on slicing (RNA silencing cycle) through molecular dynamics simulation analysis, and its subsequent statistical analysis. Those analyses revealed different structural conformations of the RNA duplex in Ago2, depending on Mg^2+^ concentration. We also demonstrate that cation effects on Ago2 structural flexibility are critical to its catalytic/functional activity, with low [Mg^2+^] favoring greater Ago2 flexibility (e.g., greater entropy) and less miRNA/mRNA duplex stability, thus favoring slicing. The latter finding was supported by a negative correlation between expression of an Mg^2+^ influx channel, TRPM7, and one miRNA’s (miR-378) ability to downregulate its mRNA target, *TMEM245*. These results imply that thermodynamics could be applied to siRNA-based therapeutic strategies, using highly complementary binding targets, because Ago2 is also involved in RNAi slicing by exogenous siRNAs. However, the efficacy of a siRNA-based approach will differ, to some extent, based on the Mg^2+^ concentration even within the same disease type; therefore, different siRNA-based approaches might be considered for patient-to-patient needs.

## Introduction

RNA interference (RNAi) utilizes double-stranded RNA (dsRNA) to mediate homology-dependent gene silencing in eukaryotic cells [1], [2]. RNAi requires two biological entities: small RNAs and the RNA-induced silencing complex (RISC) [1], [3], [4]. Two well-known small RNAs are microRNAs (miRNAs) and short interfering RNAs (siRNAs). These processed small RNAs are denatured, and the “guide strand” loaded onto the RISC, which then silences target RNAs based on their sequence complementarity to the guide strand [5]. In particular, the Argonaute 2 (Ago2) protein, a crucial component of the RISC complex, possesses slicer activity to catalyze destruction of target mRNAs [1], [2].

RNAi is increasingly being recognized as a potent therapeutic strategy [6] both for diseases and drug-resistance [7], based on its silencing of specific target mRNAs. Also, RNAi can specifically target all annotated protein-coding genes [2]. However, the silencing efficacy for target RNAs by RNAi (*e.g.*, siRNAs) varies widely, depending on thermodynamic stability, target accessibility, structural features, and position-specific determinants [2]. In addition to sequence similarity studies [5] between miRNAs and target mRNAs, recent crystallography and NMR studies [8]–[15] demonstrate 3-dimensional physical interactions between small noncoding RNAs and Ago2. Furthermore, there is high conservation between human and prokaryotic Ago protein structures [16], [17], and several recent structural studies have revealed that the divalent cation Mg^2+^ is crucial for miRNA biogenesis [18] and cleavage of its target mRNAs [8], [19]. Also, recent structural and computational studies [8], [20] revealed that inner Mg^2+^ ions inside Ago complex play an important role in RNA silencing by forming critical hydrogen bonds. This Mg^2+^ requirement argues that RNAi has another level of regulation (*i.e.*, cation concentration,) on its silencing efficacy of target RNAs.

Recent reports [19], [21] have also shown that [Mg^2+^] affects miRNA/target mRNA binding patterns in highly (or perfectly) complementary duplexes. In these studies, the cleavage activity of highly complementary (near perfect) binding patterns negatively correlated with [Mg^2+^], with higher [Mg^2+^] corresponding to lower efficacy in silencing of target RNAs. That finding would suggest that the high-complementary binding pattern generally observed between siRNAs and their targets would prefer low [Mg^2+^] over high [Mg^2+^] [22]. Such mechanistic understandings might improve siRNA function as an emerging therapeutic strategy [23], in conjunction with better understanding of the structure of Ago2, a protein involved in both miRNA target recognition and siRNA target recognition and destruction [24].

Cation effects have been examined primarily in studies of protein folding kinetics, all-atom computational simulations, and conformational states of RNA folding [25], [26]. However, current studies of ion effects have not been extended to small RNA-mediated target regulation, and it is not clear whether divalent cations (such as Mg^2+^) could affect small noncoding RNA-mediated target mRNA destruction by their modulation of ion-RNA duplex-Ago2 interactions. These effects could represent a new layer of regulation of target cleavage efficacy by specific siRNAs/miRNAs, thus affecting the various biological processes mediated by their target transcripts [27], [28]. Importance of [Mg^2+^] in miRNA processing is also shown in TRPM7 (transient receptor potential cation channel, subfamily M, member 7), being a lethal to HEK293 cells [29]. TRPM7 plays an essential role in vascular smooth muscle cell growth [30], a process well-associated with miRNA activity [31]. As RNAi strategies are increasingly being investigated for therapeutic purposes (considerations as enzymatic efficiency), “off-target” effects and triggering of interferon responses still remain to be major hurdles [2].

In the present study, we provide a mechanistic framework for Mg^2+^ effects not only on ion-RNA interactions, but also on the whole RISC complex (*i.e.,* the RNA duplex, Mg^2+^, Ago2, and water environment). We further provide structural insight into miRNA-mRNA binding and suggest potential usage of our structural biochemical results for translational applications. In particular, high complementary binding between a miRNA and its target showed better Ago2 slicing efficacy in the presence of low [Mg^2+^] versus high [Mg^2+^] [8], [19]. Based on our all-atom simulation of Ago2, cations, and water, we observed that differences in cation concentration induced distinct structural changes in the miRNA-mRNA duplex and the Ago2 protein. Energy profiles of miRNA-mRNA duplexes showed that target splitting was highly dependent on [Mg^2+^], with high concentrations favoring low duplex energy (thus indicating stabilization of the duplex structure). In contrast, we found that low Mg^2+^ concentrations favor structural flexibility of the miRNA-target RNA duplex, a condition necessary for the kinetic transitions of miRNA-target RNA slicing. Finally, we discuss the potential use of our results for biomedical siRNA therapeutics, which are physiologically affected by Mg^2+^ ions (even within the same disease subtype).

## Results

### Structural changes of RNA duplexes and Ago2 protein, based on Mg^2+^ concentration

**Figure 1:**
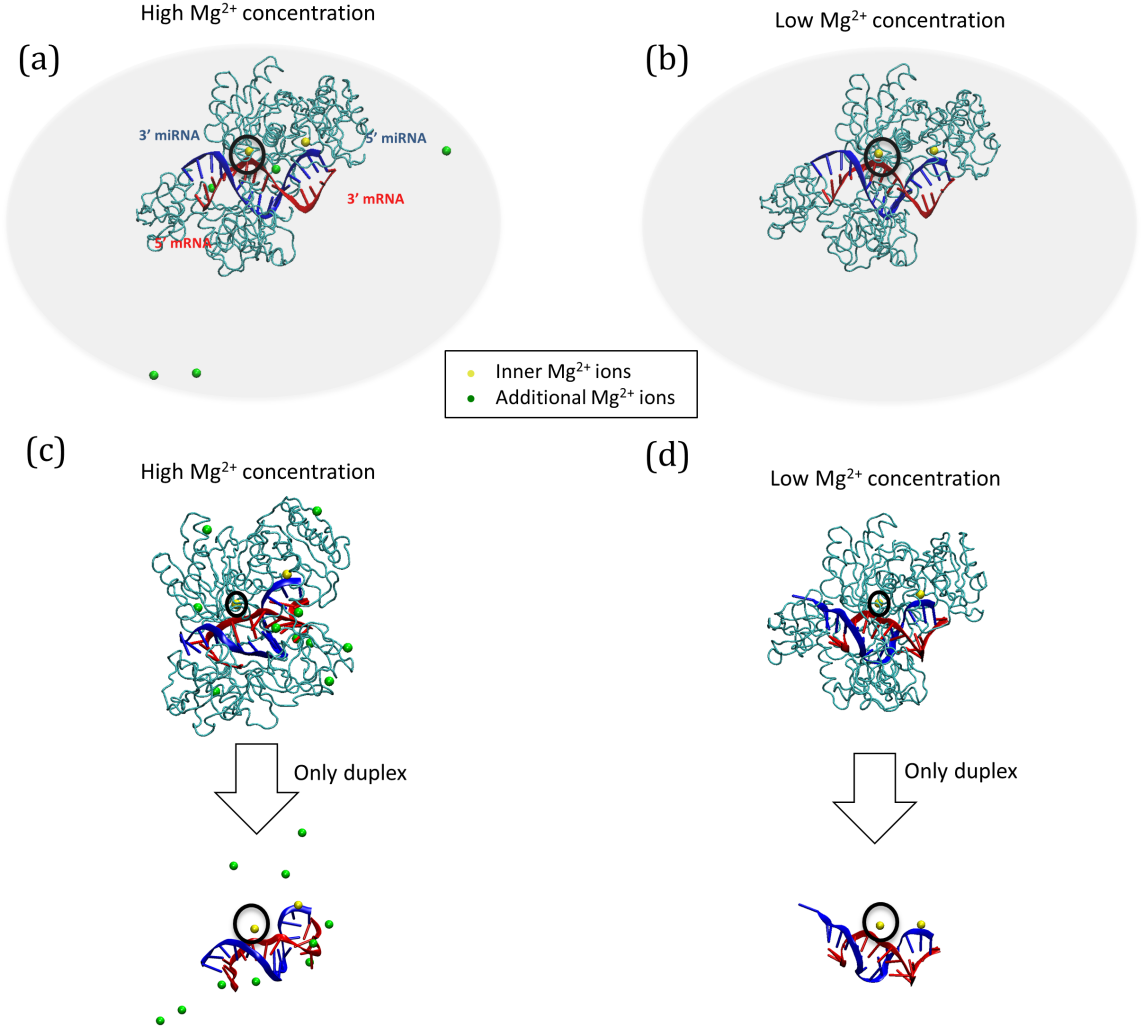
Three-dimensional structures of the miRNA-mRNA-Ago2 complex. The initial Ago2 structures under high and low concentrations of Mg^2+^ ions (Ago-high and Ago-low) are presented in (**a**) and (**b**), respectively. The final structures of the complex and the duplex after 10 ns of simulation are shown for high (**c**) and low (**d**) concentrations of Mg^2+^ ions. Two replicates (HM4 for Ago-high and LM2 for Ago-low complex) are used for drawing. The components of the complex are the Ago2 protein (cyan tube), mRNA (red strand), the miRNA (target RNA) (blue strand), inner Mg^2+^ ions (yellow spheres), and outer Mg^2+^ ions (green spheres, only in Ago-high). The black circle represents the Mg^2+^ ion in the catalytic region interacting with the target RNA (red strand). In the miRNA-mRNA duplex, the bases are drawn as tube models. The periodic box for the computational simulation is not shown.

We generated initial molecular dynamics-simulated structures [32] (along with the water environment, for enhancing biological reality) to characterize the thermodynamic features of a miRNA-mRNA duplex with its cognate protein, Ago2, under high (“Ago-high”) and low (“Ago-low”) Mg^2+^ ion concentrations (see Materials and Methods for details). We also modeled the mRNA (hereafter described as target RNA) of the RNA duplex to be perfectly complementary to the miRNA. The initial structures generated for Ago-high and Ago-low are shown in Fig. 1a and Fig. 1b, respectively. For the Ago-high environment, the initial structure of the RNA duplex and Ago2 complex accommodated 10 Mg^2+^ ions, in addition to 2 Mg^2+^ ions within the Ago2 catalytic site. For comparison, Ago-low was defined as the structure devoid of the 10 additional Mg^2+^ ions. For the two systems, Ago-high and Ago-low, we generated four replicates with different initial conditions: HM1-HM4 for Ago-high, and LM1-LM4 for Ago-low. From each replicate, molecular dynamics simulations were performed to observe structural changes and to examine how additional Mg^2+^ ions affected both the Ago protein and the miRNA-target RNA duplex stability (see Fig. 1 and Fig. S1 in File S1 for structural visualization (of the two systems) and the molecular dynamics simulation scheme, respectively).

**Figure 2:**
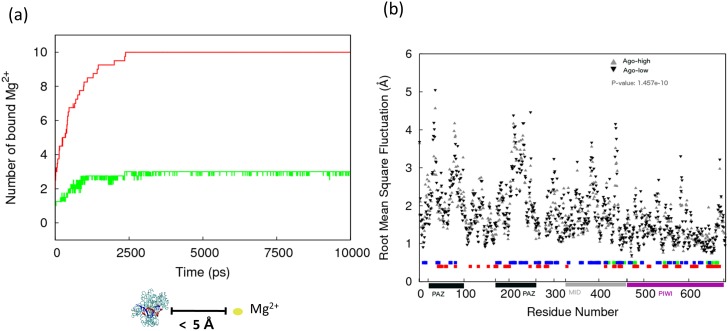
Number of Mg^2+^ ions binding to the miRNA-mRNA-Ago2 complex during the simulation of Ago-high and Ago-low, and root mean square fluctuations (RMSFs) of Cα positions. (**a**) We inspected average binding of the 10 additional Mg^2+^ ions to Ago-high during simulations of the four replicates (HM1–4). The red line (indicated as whole) represents the number of Mg^2+^ ions that bind to the whole complex (miRNA-target RNA duplex and Ago2 protein) (less than 5 Å). The number of Mg^2+^ ions that bind the duplex (less than 5 Å) are represented by green lines. Within 2,500 picoseconds (ps), three Mg^2+^ ions bound the duplex in all the replicates. (**b**) Root mean square fluctuations (RMSFs) of Cα positions by residue number in the two systems: Ago-high (grey triangle) and Ago-low (black inverted triangle) complexes. To compare the protein flexibilities of the two systems in terms of ion-binding residues and duplex-binding residues, we considered Ago-high as the reference. The RMSF plot was drawn from all replicates of Ago-high and Ago-low complexes. The red boxes right above the x-axis indicate the amino acid residues that interact with the RNA duplex of Ago-high. The green boxes right above the x-axis indicate the amino acid residues that bind to the inner 2 Mg^2+^ ions of Ago-high. The blue boxes right above the x-axis indicate the amino acid residues that interact with the 10 additional Mg^2+^ ions of Ago-high. The four horizontal rectangles beneath the x-axis indicate the three domain regions: PAZ (20–100 a.a, 170–260 a.a), MID (326–462 a.a), and PIWI (463–678 a.a). Under the Ago-high condition, the additional Mg^2+^ ions interacted with all the three domains, as did the duplex. Interestingly, the inner two Mg^2+^ ions interacted with only two domains (PIWI and MID), but not the PAZ domain, indicating that intracellular Mg^2+^ ions (blue boxes) have much more interactions with the amino acids than the PIWI-bound inner 2 Mg^2+^ ions (green boxes). Also, the higher intracellular [Mg^2+^] (Ago-high) showed smaller RMSFs in all the domains, compared to low intracellular [Mg^2+^] (Ago-low). The PIWI domain, responsible for target cleavage, revealed smaller RMSF differences between high [Mg^2+^] (Ago-high) and low [Mg^2+^] (Ago-low) over the other two domains, indicating that additional intracellular Mg^2+^ ions outside the complex do not affect PIWI domain structure, but diminish structural flexibility of the other two domains (especially the PAZ domain).

To evaluate the structural equilibrium of the Ago2 protein during simulation, root mean square deviations (RMSDs) of the various Ago2 alpha carbon (Cα) positions were obtained every 1 picosecond (ps) after its initial state. As shown in Fig. S2 in File S1, both systems (Ago-low and Ago-high) were rearranged and then stabilized in a simulation of ∼1 nanosecond (ns). In the Ago-high complex, all of the additional Mg^2+^ ions were bound in the whole (RNA duplex and Ago protein) complex in ∼2.5 ns (Fig. 2a, red line). Because of the positive charges of the Mg^2+^ ions, the ions easily interacted with the negative charges of the amino acid residues (Asp and Glu) and those of the RNA phosphate groups. We inspected the amino acids close to (equivalently, interacting with) the additional intracellular 10 Mg^2+^ ions (located outside Ago2 protein; Fig. 1c, green spheres), the inner PIWI-bound 2 Mg^2+^ ions (located inside Ago2 protein; Fig. 1c, yellow spheres), and the miRNA-target RNA duplex in the final structures of the Ago-high complex. While the additional Mg^2+^ ions located outside the Ago2 protein interacted with the various residues throughout all the domains, the inner PIWI-bound Mg^2+^ ions interacted with limited number of residues only within the MID and PIWI domains (Table S1 in File S1). We also listed the intermolecular contacts near the RNA duplex in the duplex-binding channel, according to high and low Mg^2+^ concentrations (*i.e.*, Ago-high, Ago-low respectively) (Table S2 in File S1. In Ago-high, a maximum of 2∼3 of the 10 additional Mg^2+^ ions were allowed to bind to the miRNA-target RNA duplex (Fig. 2a green line; and Table S2 in File S1). Furthermore, these ions quickly and preferentially approached and bound the 5′ terminal region of the miRNA (equivalent to the 3′ terminal region of the target RNA) of the negative phosphate groups of the RNA duplex early in the simulations (Figs. 1c and 2a). In the final miRNA-target RNA duplexes (Figs. 1c and 1d), the pairings in the central region were considerably conserved in the Ago-high complex, but not in the Ago-low complex. Meanwhile, in the Ago-low complex, the base pairings of the RNA duplex were not conserved in the 3′-miRNA terminal region (equivalent to the 5′-mRNA terminal region) (Fig. 1d), indicating that this loose region may initiate dissociation (for turnover) after target RNA cleavage involving the inner Mg^2+^ ion (yellow sphere in the black circle in Fig. 1d; cleavage region of mRNA).

**Figure 3:**
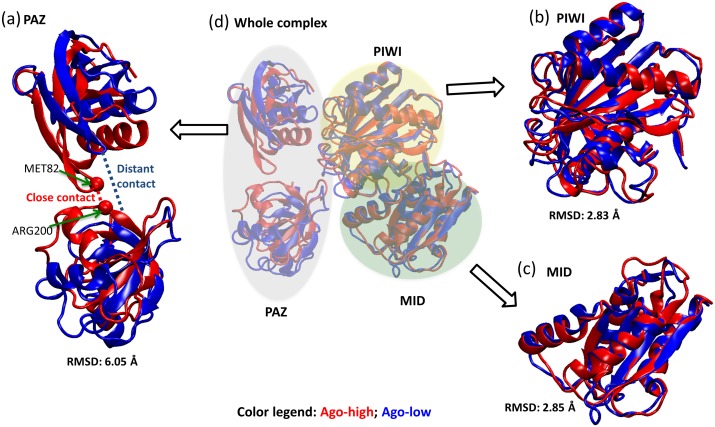
Structural comparison of Ago proteins between high [Mg^2+^] (Ago-high; in red cartoon) and low [Mg^2+^] (Ago-low; in blue cartoon). (**a**) Superimposition of two subdomains of PAZ. The similarity score, measured by root mean square deviation (RMSD) of superimposed heavy atoms (all atoms except hydrogen), was 6.05 Å. Note that the PAZ domain consists of the two separated domains. These two subdomains were closer each other in Ago-high structure compared to Ago-low structure. In a final structure of the Ago-high replicates, Met82 and Arg200 were the closest amino acid pair between the subdomains. Also, the closest distances of the two subdomains in Ago-low kept changed from 2 to 12Åthroughout all time points, while those of Ago-high remained to be constant (∼2Å nearly in all the time points (see Fig. S3 in File S1). It indicates the PAZ domain under high [Mg^2+^] come to be rigid, resulting in resistance to structural flexibility. (**b**) Superimposition of PIWI domains of Ago-high and Ago-low were depicted, and RMSD was 2.83 Å. (**c**) Superimposition of MID domains of Ago-high and Ago-low were depicted, and RMSD was 2.85 Å. (**d**) Superimposition of whole Ago2 proteins of Ago-high and Ago-low were depicted and RMSD was 5.62 Å.

To examine structural differences due to Mg^2+^, we measured the conformational similarity of the final structures of the Ago-high and Ago-low systems (Fig. 3) by superimposition. In the whole Ago2 complex, the additional Mg^2+^ ions in Ago-high induced an average of 5.62 Å RMSD in structure (Fig. 3d). Examination of specific Ago2 domains (MID, PAZ, PIWI) showed that two PAZ subdomains had close contacts with each other in Ago-high compared Ago-low, in terms of physical closeness (Fig. 3a and Fig. S3 in File S1). Also, the PAZ domain underwent a more drastic structural difference (6.05 Å RMSD; Fig. 3a) in Ago-high, compared to the PIWI (2.83 Å RMSD, Fig. 3b) and MID (2.85 Å RMSD, Fig. 3c) domains. Therefore, most of the Ago2 structural differences induced by Mg^2+^ levels occurred in the PAZ domain (6.05 Å, Fig. 3a), while the changes of the other two domains were relatively small: PIWI (2.83 Å RMSD) and MID (2.85 Å RMSD). The tight physical proximity (red-dotted line in Fig. 3a; Met82-Arg200) of the two PAZ subdomains in Ago-high, compared to Ago-low, induced a large structural change. This physical closeness (Fig. S3 in File S1) between the two PAZ subdomains could cause the Ago2 protein, in the presence of high Mg^2+^, to be resistant to the structural changes elicited by thermal energy fluctuations, thus slowing down Ago2 catalytic activity, due to structural inflexibility. In contrast, the weak subdomain-interaction under low [Mg^2+^] conditions could render the Ago2 protein to be more structurally flexible (fitting to thermal fluctuation), possibly conferring enhanced catalytic activity.

Comparison of high (Fig. 1c) versus low (Fig. 1d) Mg^2+^ concentrations further showed the terminal ends of the duplex to be uncoiled, the extent of which was affected by Mg^2+^ levels. Thus, we further dissected the energy of local regions of the duplex for the different Mg^2+^ concentrations, using statistical analysis.

### Energy difference between the duplexes at high and low Mg^2+^ concentrations

To investigate the effect of cations on the miRNA-target RNA duplex within the Ago2 protein, we measured the average base-pairing energy between the miRNA and the mRNA (target RNA) as a function of simulation time (Fig. S4 in File S1). The average base pairing energy reached a plateau after 1 ns of simulation. The average base pairing energies of the overall duplexes from the two systems (Ago-high and Ago-low) are tabulated in Table 1 (see the bottom row). Moreover, to observe the local energies (equivalent to the local structures) of the two duplexes, we divided the miRNA-mRNA duplex into three factor levels, shown in Table 1, (Region1: mRNA 5′-terminal region and miRNA 3′-terminal region; Region2: duplex central region; and Region3: mRNA 3′-terminal region and miRNA 5′-terminal region) and measured the local energy patterns between Ago-high and Ago-low, as shown in a 3 by 2 contingency table (numerals in italic in Table 1) (see Materials and Methods). We then compared each level of factor Group (in a given level of factor region) by using contrasts [33] to observe local regional energy differences between Ago-high and Ago-low. As shown in Table 1, we observed 3-dimensional differences in the energy/structure states of the two 2-dimensionally identical miRNA-target RNA duplexes in the Ago-high and Ago-low complexes. Moreover, the energy differences in the three local regions between Ago-high and Ago-low were statistically significant (bold numerals in Table 1, last column). Thus, these results further describe the 3-dimensional characteristics of the two systems, Ago-low and Ago-high.

**Table 1:** Average base pairing energy profile of the RNA duplexes of the two systems (Ago-high and Ago-low).

Factors		Group		ΔE^a^	P-valueof ΔE
Region		Paring energy(Ago-low)	Paring energy(Ago-high)		
Region1	5′-**CAGU**CUGAUAAGCUA-3′(mRNA)	*−3.346±3.551*	*−5.042±4.733*	1.696^a^	**0.00000** b
	3′-**GUCA**GACUAUUCGAU-5′(miRNA)				
Region2	5′-CAGU**CUGAUAA**GCUA-3′(mRNA)	*−4.440±2.945*	*−6.313±2.355*	1.873^a^	**0.00000** b
	3′-GUCA**GACUAUU**CGAU-5′(miRNA)				
Region3	5′-CAGUCUGAUAA**GCUA**-3′(mRNA)	*−4.979±3.284*	*−3.821±3.671*	*−*1.158^a^	**0.00000** b
	3′-GUCAGACUAUU**CGAU**-5′(miRNA)				
Average whole duplex pairing energy		Ago-low	Ago-high	ΔE^a^	P-value of ΔE
		*−*4.292±1.420	*−*5.310±2.317	1.018^a^	<2.2e-16^c^

The average energy profile of the local regions in the duplex was summarized based on the all-atom simulation of the miRNA-target RNA-Ago complex in an implicit water environment with different Mg^2+^ concentrations (Ago-low and Ago-high). We divided the duplex into the three local regions, depicted as “Region1” (mRNA 5′-terminal region and miRNA 3′-terminal region; bold region in the duplex), “Region2” (the middle region of the duplex; bold region in the duplex), and “Region3” (mRNA 3′-terminal region and miRNA 5′-region; bold region in the duplex).

a Energy difference from Ago-low to Ago-high (the energy unit: kcal/mol).

b Test of contrast in conjunction with the full linear model as described in the Materials and Methods section.

c Two sample t-test.

In the final 3D structure of the miRNA-target RNA duplex in Ago-high (Fig. 1c), base pairing was not disrupted in the central region, and the base pairing conformation was conserved throughout the simulations. In particular, the central regions of both complexes (Ago-high and Ago-low) showed lower base pairing energies than their corresponding overall base pairing energies (Table 1). In addition, the Ago-high central region indicated its miRNA-mRNA duplex structure to have greater energy than that of Ago-low. In other words, whereas the all-pairing energy of Ago-high was lower than that of Ago-low by 1.018 kcal/mol, the central region base pairing (Region2 in Table 1; 5∼11 bps from the 5′ directions of both the miRNA and the target RNA) of Ago-high was lower than that of Ago-low by 1.873 kcal/mol (Table 1). This difference indicates that the central region (Region2 in Table 1) of the Ago-high complex becomes more rigid. In both models (Ago-high and Ago-low), the energies of the terminal regions (Region1 and Region3 in Table 1) of the duplex were generally higher than, or at least approximately equal to, those of the central region (Region2 in Table 1). Thus, the terminal regions harboring loose base parings and uncoiled conformations are consistent with the configurations shown in Figs. 1c and 1d.

Comparison between the global energy profiles of Ago-low and Ago-high indicated the average base pair energy in the Ago-high complex to be significantly (1.018 kcal/mol, p-value<2.2e-16 in Table 1) lower than that in the Ago-low complex, thus suggesting greater thermodynamic structural stability under high [Mg^2+^] conditions.

### Conformational entropy and RMSF (structural flexibility)

Because catalytic activity generally depends not only on structural stability but also on flexibility [34], we also quantified structural flexibility. Proteins experience a series of conformational changes during their function (or catalytic activity); these are highly dependent upon structural flexibility [34], [35]. For example, Ago2 undergoes several conformational steps (RNA loading, nucleation, propagation and slicing, and target release) during the RNA slicing cycle [36]. Consequently, we numerically quantified the structural flexibilities (using root mean square fluctuation (RMSFs)) of the two systems, Ago-high and Ago-low (Fig. 2b), in order to see flexibility differences between the two systems.

To numerically quantify structural flexibility, we inspected how additional ions affect global Ago2 protein conformation by comparing the Cα positions between Ago-high and Ago-low structures. The Ago2 protein Cα residues (red-filled boxes in bottom of Fig. 2b) bound to the RNA duplex had smaller RMSF values (*i.e.*, less flexibility) in both Ago-low and Ago-high. However, Ago-high residues (blue-filled boxes in the bottom of Fig. 2b) interacting with the additional 10 Mg^2+^ ions showed both flexibility and rigidity. Overall, the structural fluctuation of Ago-high was less than that of Ago-low. In fact, the paired RMSF differences between Ago-low and Ago-high for the Cα positions were significantly greater than zero (paired t-test: p-value 1.46e-10). This finding indicates that a higher Mg^2+^ concentration corresponds to more rigidity in the Ago2 movement. This Ago-high rigidity in protein motion would tend to oppose conformational changes required for the silencing cycle, resulting in low cleavage efficacy (*i.e.*, slow turnover).

To quantify the flexibilities of Ago-high and Ago-low, we examined the conformational entropy of the backbone by dividing RMSFs into two conformational states: ordered (RMSF<0.3) and disordered (RMSF≥0.3). Subsequently, the conformational entropy S of the backbone was obtained from the probabilities of the states (see Materials and Methods). The greater S value indicates flexibility, meaning that the protein backbone resides in a disordered state. The S for Ago-low was 0.293, and that for Ago-high, 0.246. Those entropies indicate that higher [Mg^2+^] increases the rigidity of the Ago protein backbone, consistent with the previous qualitative analysis. The entropy analysis of the two systems indicates that entropy favors low over high Mg^2+^ concentrations. Thus, the high entropy for the low Mg^2+^ concentration could be beneficial to a series of conformational changes.

We inspected Ago2 structural flexibility relating to intracellular Mg^2+^ ion gradient (*i.e.*, 2, 4, 6, and 8 intracellular Mg^2+^ ions). We generated the additional systems according to the number of intracellular Mg^2+^ ions (2, 4, 6, and 8). The same molecular dynamics simulations set-up used in Ago-high (equivalently, 10 intracellular Mg^2+^ ions) and Ago-low (equivalently, 0 intracellular Mg^2+^ ion) was applied to the additional systems. Each system had four replicates. We show in Fig. S5 in File S1, the conformational entropy such as Ago protein flexibility loss (*R^2^*: 0.738), adding evidence that higher Mg^2+^ concentration cause rigidity in protein. This induced rigidity by additional intracellular Mg^2+^ ions could slow down conformational changes [34], [35] that are inevitable for multiple transitions in the RNA silencing steps, *i.e.*, guide strand loading, nucleation, propagation, slicing (equivalently, cleavage), and target release [8], [36], [37]. Thus, high Mg^2+^ can cause rigidity and causing delay in the RNA silencing cycle.

Combining the energy and entropy analyses indicated that greater numbers of Mg^2+^ ions could stabilize, in particular, the central region of the miRNA-target RNA duplex. The ions stabilize the duplex structure but simultaneously cause fewer degrees of freedom, which govern conformational changes. Given the low RNA silencing efficacy under high [Mg^2+^] conditions [19], the degrees of freedom (higher conformational entropy) of the Ago2 protein (under low [Mg^2+^] conditions) could be more favorable for conformational changes, than the RNA duplex structural stability, during the slicing cycle.

### Expression change of an mRNA target by miR-378 according to Mg^2+^ importer gene expression

**Figure 4:**
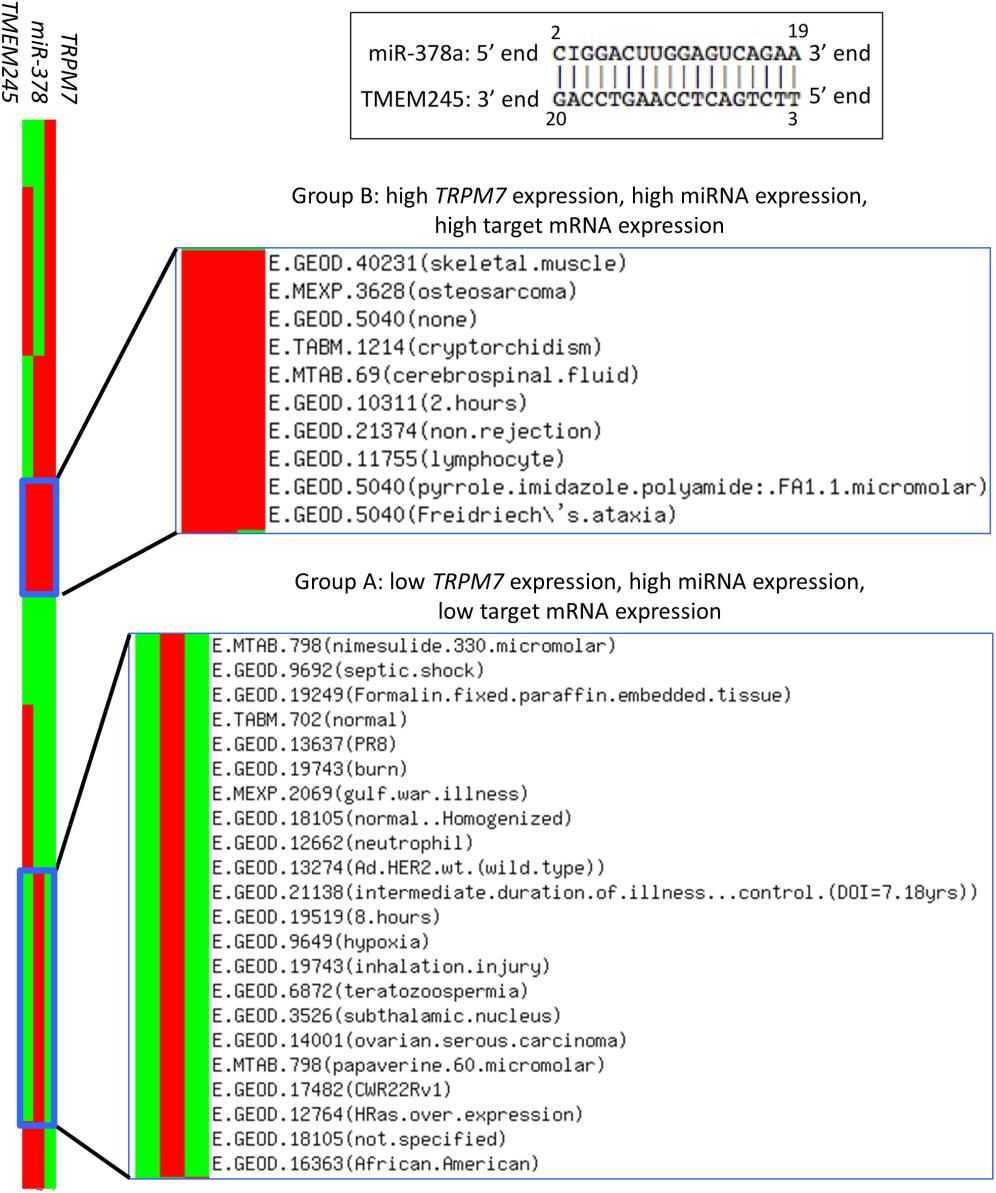
Expression heatmap of the cellular Mg^2+^ importing gene (*TRPM7*), miR-378, and the miR-378 target *TMEM245*. The expression of miR-378 and its target *TMEM245* (high complementary binding; see the upper black box) was associated with *TRPM7* expression (left-most panel). From the Expression Atlas [40], we obtained 95 experiments showing significant differential expressions of *TRPM7*, miR-378, and *TMEM245*. The red and green colors indicate high and low expression in a given experiment, respectively. Group A represents studies showing high miRNA expression, low target mRNA expression, and low *TRPM7* expression. Group B represents studies showing high miRNA expression, high target mRNA expression, and high *TRPM7* expression. The ArrayExpress accession numbers and their experiment descriptions in Groups A and B are represented in the row names.

**Figure 5:**
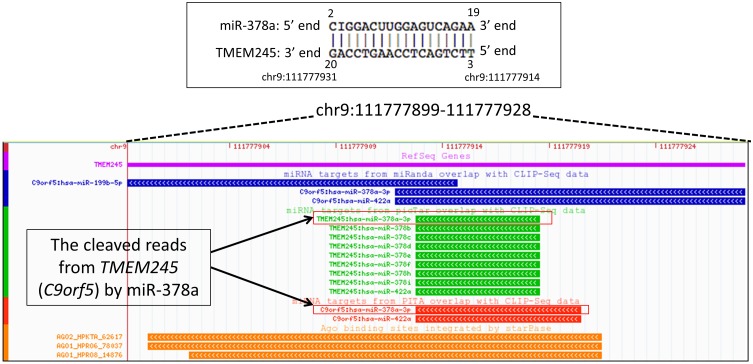
CLIP-Seq evidence of *TMEM245* and miR-387. To see *TMEM245* cleavage by miR-378a, CLIP-Seq supported miRNA-target mRNA database was inspected around the binding site (upper rectangular box; genomic coordinate also provided). The red box in the browser screen-capture represents the fragments from the binding site region. The genomic coordinates were GRCh37 (UCSC hg19 assembly).

To validate our structural study, we further inspected possible biological effects of Mg^2+^ on target mRNA slicing by a miRNA. Mg^2+^ ions are imported into the cell by TRPM7, an Mg^2+^ channel protein [38], [39]. Thus, high expression of *TRPM7* correlates with a high intracellular [Mg^2+^] [38], [39]. In Fig. 4, according to *TRPM7* expression, a target mRNA (*TMEM245*) expression was altered by miR-378 in 95 various experiments from the Expression Atlas [40] (www.ebi.ac.uk/gxa). The binding pattern between the miRNA and its target was highly complementary (upper black box in Fig. 4). Comparing Groups A (low [Mg^2+^]) and B (high [Mg^2+^]) (in Fig. 4), we observed that, despite the high miRNA expression in both groups, the expressions of the target mRNA in Group A experiments were lower than those in Group B experiments. We also note that the public miRNA-target mRNA CLIP-Seq database [41] showed this binding site to be biologically relevant to target degradation (Fig. 5).

### Implication of siRNA therapy according to Mg^2+^ importer gene expression

Because Ago2 interacts not only with miRNAs but also with siRNAs [1], [2], [42], our analysis of the effect of cations on high complementarity duplex binding can be applied to siRNA efficacy. Furthermore, siRNAs that are designed to bind with highly complementarity to mRNA targets have been recognized as potential therapeutic tools [23], [29]. Since intracellular ion concentrations can differ, even in the same cancer subtype, we assessed expression of the Mg^2+^ channel gene, *TRPM7*, in 49 basal subtype breast cancer tumors found in the The Cancer Genome Atlas [43], [44]. The 49 basal subtype tumors were divided into 12 high *TRPM7*-expressors and 37 low *TRPM7*-expressors (Table 2). Thus, a therapeutic strategy that uses target gene knockdown via siRNAs should be carefully administrated, due to possible variations in intracellular Mg^2+^ levels (Table 2). In Table 2, we also list expression patterns of oncogenic kinases potentially targeted by siRNAs, showing this strategy to be quite feasible.

**Table 2:** Patient profile according to *TRPM7* expression in basal subtype breast cancer patients.

Basal subtype	*TRPM7* high/low	Oncogenic kinases aspotential siRNA targetgenes (%: proportion ofhighly expressed cases)
49 cases	12 cases with high*TRPM7* expression	EGFR: 75.00% cases
		CDK6: 83.33% cases
		MET: 75.00% cases
		PIM1: 75.00% cases
		ABL2: 41.67% cases
	37 cases with low*TRPM7* expression	EGFR: 91.89% cases
		CDK6: 54.05% cases
		MET: 54.05% cases
		PIM1: 43.24% cases
		ABL2: 62.16% cases

We observed *TRPM7*, Mg^2+^ ion importing protein-coding gene, was differentially expressed even in the same cancer subtype. It indicates that Mg^2+^ ion concentration is different from individual to individual and subsequently siRNA efficacy for potential oncogeneic kinases could differ. We set “Select Cancer Study”, “Select Genomic Profiles”, “Enter a z-score threshold”, and “Select Patient/Case Set” to “Breast Invasive Carcinoma (TCGA, Nature 2012)”, “mRNA Expression z-Scores (microarray)”, 1.0, and “PAM50 Basal”, respectively, in cBioportal.org. The z-score threshold is used to identify high or low-regulated genes. According to high or low *TRPM7* expression, the patients were divided to 12 high *TRPM7*-expressed cases and 37 low *TRPM7*-expressed cases (total 49 patients). The efficacy of siRNA for the target genes could be lower in the patients with high *TRPM7* expression, whereas the RNAi therapeutic intervention for the target genes could be more favorable for the patients with low *TRPM7* expression. For example, siRNAs targeting *EGFR* would be preferable for 91.89% of the *TRPM7* down-regulated patients, while the siRNAs would not be preferable for the majority (75%) of *TRPM7* up-regulated patients. More comprehensive data for other oncogenic kinases are in Tables S4–S37 in File S1.

## Discussion

**Figure 6:**
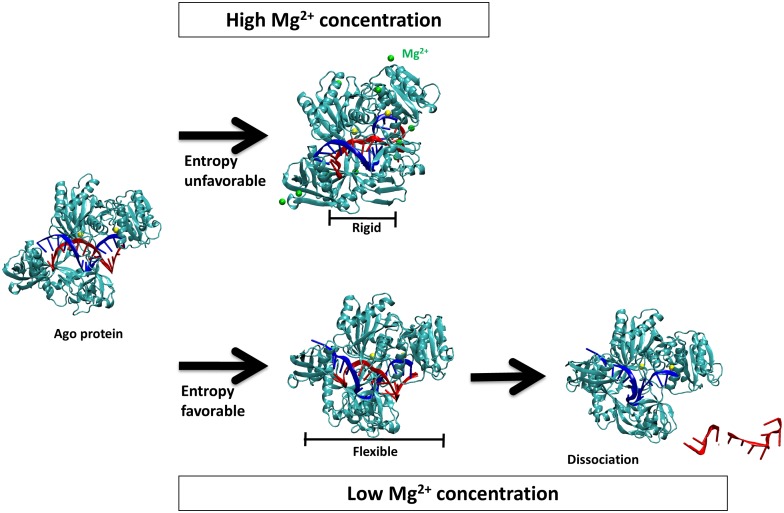
Conformational state scheme from the miRNA silencing catalytic reaction. The left miRNA-target mRNA mode, a degenerate state, is split to previously unknown conformation states by the [Mg^2+^]. Under low [Mg^2+^] (*lower panel*), the protein has more flexibility (*i.e.*, disordered with favorable entropy), but the duplex has unfavorable energy. Thus, the duplex (blue: miRNA; red: target RNA) is easily dissociated into two single strands after cleavage, and the cleavage products more readily exit the flexible Ago protein. Under high [Mg^2+^] conditions (*upper panel*), the duplex is energy-favorable, and the Ago protein is less flexible (*i.e.*, more ordered with unfavorable entropy). Thus, the duplex slicing could be slower, and the cleavage products could be less able to exit the Ago protein.

Our study shows that molecular dynamics simulations provide a solid computational explanation of how the miRNA-target mRNA-Ago complex might depend on [Mg^2+^]. For this purpose, we quantified the energy and entropy in full systems (Ago-low and Ago-high) using trajectory analysis of molecular dynamics simulations. Stabilization of the Ago protein and the mRNA-miRNA complex at high [Mg^2+^] could weaken the slicing cycle due to the loss of degrees of freedom (structural flexibility or entropy) that are necessary for slicing cycle-associated conformational changes. In other words, our study reveals that Mg^2+^ ions split the degenerate energy level of a high-complementary binding pattern (between miRNA and target RNA) into previously unexplored energy (or 3-dimensional configuration) states (Fig. 6). Low [Mg^2+^] confers lower duplex stability, while also allowing conformational changes of the Ago2 protein, during the slicing cycle. Thus, our results strongly suggest that ion concentration is critical for miRNA-target RNA silencing activity by providing a balance between the RNA duplex structural stability and the Ago2 protein structural flexibility.

At low ion concentrations, a weaker interaction in the 3′ end of the miRNA (Region1), compared with that in the other regions (Region2, Region3), was observed during the molecular dynamics simulation (Fig. 1d and Table 1). This finding could demonstrate the preference of the miRNA (or guide strand) 3′ region as an initiation point for dissociation (target release step), consistent with previous biochemical studies [16], [21], [42]. In the same context, comparing the miRNA 3′ regions of the two models (stronger interaction in Ago-high than in Ago-low) implies that under the high ion concentration dissociation of the miRNA 3′ region from Ago2 is unfavorable, leading to a slower target release step, followed by the next silencing cycle.

Our computational analysis emphasizes intracellular Mg^2+^ ions rather than the inner PIWI-bound Mg^2+^ ions, providing additional insight into RNAi silencing. We note that the Ago protein in the RNAi complex plays a critical role in a series of steps; i) guide strand loading, ii) nucleation, iii) propagation, iv) slicing (equivalently, cleavage), iv) target release [8], [36], [37]. Of these steps, the propagation step (in target recognition) between the guide strand and the target strand requires the release of the guide strand 3′ end anchored in the PAZ domain pocket [8], [37]. This release results from securing the RNA duplex-binding channel in Ago [8], and also induces a PAZ domain structural change [8], [37], followed by the cleavage step. In other words, the target RNA cleavage step, involved in the inner Mg^2+^ ions and RNase H-like fold in the PIWI domain, requires 3′-end release of the guide strand. During the Ago-mediated slicing cycle, not only the PIWI and MID domains, but also PAZ [8], [36] domain is crucial.

Recent computational and structural studies [8], [20], [37] provide significant insight into the roles of the “inner Mg^2+^ ions” (PIWI-bound Mg^2+^ ions inside the Ago) on the three domains (PIWI, MID, PAZ) and RNA duplex. Ma *et al.* [20] intensively inspected the Ago2 complex containing RNA duplex in terms of the “inner Mg^2+^ ions” (inside the Ago) by molecular dynamics simulations. They concluded that the inner Mg^2+^ ions regulate all three domains (PIWI, MID, PAZ) coordinately, as well as miRNA-target RNA interactions. In contrast, our study proves to show biologically realistic settings through intracellular Mg^2+^ ions (*i.e.*, high and low [Mg^2+^], corresponding to Ago-high and –low, respectively) outside the Ago2 protein. In fact, human intracellular free [Mg^2+^] levels range from 0.25 to 1 mM [45]. Of interest, our result indicated that the 10 additional intracellular Mg^2+^ ions in Ago-high induced greater structural RMSF decrease of PAZ than those of the other two domains (Fig. 3). Due to the role of PAZ [8], [37] on the guide strand 3′-end release (during the propagation step) prior to the target cleavage step, the drastic loss of structural flexibility of PAZ by the additional intracellular Mg^2+^ ions is likely to affect the transition to the target cleavage step, possibly slowing down overall RNAi cycle efficiency under high intracellular [Mg^2+^] conditions (*i.e.*, Ago-high). Considering that protein structural flexibility is a key for enzymatic kinetics [34], [35], our result implies that the dependence [19], [21] of Ago2-mediated RNA silencing efficiency on intracellular [Mg^2+^] is due to a loss of structural flexibility and equivalently, a loss of entropy.

Improper function of membrane proteins, including TRPM7 [29], that control ion influx may dysregulate miRNA-mediated target mRNA cleavage, resulting in pathogenesis via disruption of the mRNA-miRNA cleavage mechanism regulated by [Mg^2+^]. In addition, our results imply that siRNAs with high complementarity would not function effectively in a high [Mg^2+^] environment. Under high [Mg^2+^] conditions, introducing an internal mismatch to the central region of the siRNA [22] could be a strategy for disrupting its tight base pairing with its target, thus increasing entropy within the duplex.

Highly complementary binding patterns between miRNAs and their target mRNAs depends on the concentration of Mg^2+^ ions in a cell system. This dependence indicates that Mg^2+^ chemistry affects miRNA-mediated target mRNA degradation by modulating the slicing cycle (*i.e.*, dissociation of duplex after cleavage), leading to dys-regulation of multiple target mRNAs. In fact, the human body contains various intracellular free [Mg^2+^] levels ranging from 0.25 to 1 mM [45], and target mRNA expression could be differentially affected. In addition, our results suggest that even for the same pathology subtype (breast cancer), siRNA-based therapeutic strategies [7], [46] could be selectively administered based on the specific physiological conditions (e.g., [Mg^2+^], siRNA/target complementarity, *etc*.) of patients.

However, our study has a limitation of dealing with missing amino acids in the PDB ID 3HK2 structure. Although our computational analysis should be carefully interpreted, our analysis is one of few studies of the effects of intracellular cations (outside Ago2 complex) on Ago2 flexibility, in RNA silencing. Another limitation of our study is consideration of the effects of water on cleavage. Even though our molecular dynamics simulations considered a GBSW (see Materials and Methods) implicit water environment for water effects, our study did not introduce water molecules explicitly into the Ago2 structures. Thus, water effects on the binding poses were not evaluated in this study.

In summary, we have quantitatively demonstrated multiple thermodynamic states of RISC-associated RNA duplexes and RISC protein structural flexibility, in association with Mg^2+^ concentration. Those differential states suggest that consideration of individual-to-individual variations in ion chemistry could modulate the efficacy of RNAi-based therapeutic interventions, even in the same disease subtype.

## Materials and Methods

### Structural changes of the RNA duplex and Ago2 protein correlated with Mg^2+^ concentration

The Ago2 protein-RNA complex used in our analysis was composed of four parts: (i) Argonaute (Ago2) component (682 amino acids); (ii) mRNA (15 nucleotides); (iii) miRNA (15 nucleotides); and (iv) two Mg^2+^ ions buried in the Ago2 protein. We modeled this complex system using the biomolecular simulation tool, CHARMM [32]. We inspected available Ago protein structures in the RCSB Protein Data Bank (Table S3 in File S1) and set up our criteria for selecting the Ago protein based on: i) duplex availability of the structure; ii) duplex length; and iii) high resolution. PDB ID 3HK2 satisfied all those criteria. Human Ago2 (hAgo2) and prokaryotic Ago protein structures are strongly conserved, and 3HK2 was found more suitable than the other structures for simulating Ago2-RNA duplex formation by molecular dynamics. We designed to mimic the same experimental setting of Bartel and colleagues [19] by replacing the original RNA duplex sequences (of 3HK2) with the two same sequences (miR-21, K89). Moreover, the coordinates of the replaced duplex were the same as those of the original one. Finally, since the 15-mers were the centered region (of the target mRNA) critical to RNA silencing [19], we used 15-mers instead of 21–24-mers. The base crystal structure, 3HK2, is suitable for an all atom simulation of the miRNA-mRNA target-Ago complex because it not only has a guide strand and a target strand but also has the Mg^2+^-bound Ago protein. The sequences of the miRNA (miR-21, described in ref.[19]) and the target RNA (K89-21as, described in ref.[19]) are 3′-GUCAGACUAUUCGAU-5′ (miRNA) and 5′-CAGUCUGAUAAGCUA-3′ (target RNA), respectively. The guide and target strands in the base crystal structure were replaced with these sequences to reconstruct our initial molecular model. The initial model was fitted to the origin, and a periodic box with a volume of 152×152×152 Å^3^ was applied to prevent additional Mg^2+^ ions from exiting the Ago complex. For preparing the initial models (Ago-high and Ago-low described above), the CHARMM-gui web server (www.charmm-gui.org) [47] was used to enable us to solvate the PDB ID 3HK2 structure in the implicit solvent, and to set up the periodic boundary condition, generating CHARMM readable format.

From the initial molecular model (Ago-low complex; Fig. 1b), we generated another model having additional Mg^2+^ ions (Ago-high complex; Fig. 1a). The Ago-high complex represents high [Mg^2+^] in physiological solutions, whereas Ago-low mimics a low [Mg^2+^] environment. The high [Mg^2+^] was set to 5 mM, a concentration equivalent to 10 additional Mg^2+^ ions randomly placed in the generated periodic box. To account not only for the effects of water in a real biological condition, but also to reduce the computational burden, both complexes (Ago-high/low complexes) were simulated using an implicit solvation model, the Generalized Born model with a simple SWitch (GBSW) function [48]. The ion radii for GBSW are, in general, fitted to reproduce experimental hydration energies. However, in this study, since the GBSW radius for Mg^2+^ was not available, we used the general radius for Mg^2+^ (1.5 Å).

Molecular dynamics simulations were run for 10 nanoseconds (ns), and structures sampled every picosecond (ps) for subsequent trajectory analysis. We performed four replicate molecular dynamics simulations (with different initial atom velocities generated by random seeds) for the initial molecular models, Ago-low and Ago-high. Various structural properties were measured as a function of time, including the root mean square deviation (RMSD) of the Cα position from that in the initial Ago protein, the number of Mg^2+^ ions bound to the Ago complex, and the average base-pairing energies of mRNA-miRNA duplex. The root mean square fluctuations (RMSFs) of 664 Cα positions (18 residues are missing in PDB) were measured and separated by residue number to observe the structural flexibilities of the two (Ago-high and Ago-low) s77ystems. Each system, as mentioned earlier, had four replicates. For a given replicate, we binned the 664 RMSF values into two bins and considered each bin a conformational state. The bin with an RMSF less than 3 Å was considered an ordered state, and the bin with an RMSF greater than or equal to 3 Å considered a disordered state. Subsequently, we obtained the conformational entropy 
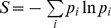
, where *p_i_* (*i* ∈ {ordered state, disordered state}) is the probability of a system being assigned to the *i-*th state.

### Determination of energy differences between Ago-high and Ago-low

To inspect the local regional energy profiles of the two systems (Ago-high and Ago-low), we divided the miRNA:target-mRNA duplex into three factor levels, shown in Table 1 (Region1: mRNA 5′-terminal region and miRNA 3′-terminal region; Region2: duplex central region; and Region3: mRNA 3′-terminal region and miRNA 5′-terminal region). We then constructed a 3 by 2 contingency table of the energy for the three regions and the two systems (Ago-high and Ago-low) to show local regional energy differences. Furthermore, we statistically tested the local regional energy differences between the two systems using a linear model based on the contingency table. We named the factor associated with the duplex local regions (Table 1) as “Region,” and the factor relating to the two systems (Ago-high and Ago-low) as “Group”. Given a local region in at a specific time point, we calculated an average base-pairing energy (summation of all the individual base- pairing energies over the local region length). The average energies were obtained throughout all the sampling time-points (every 5ps from 7ns to 10ns) from all eight models (four replicate models for each system). The average energies were used as observations for the response variable “Energy”. We fitted a full linear model, Energy = Region + Group + Region×Group, to the contingency table, to test the local average energy differences between the two systems. The significance of the linear model showed a p-value less than 2.2e-16, indicating the model to fit well with the data.

### Identification of oncogenic kinases having siRNAs

As of the time of manuscript preparation, the NCBI Probe Database [49] (ncbi.nlm.nih.gov/probe), which includes nucleic reagents that are widely used in biomedical applications, reported 169,705 antisense sequences from siRNAs and shRNAs for target genes or mRNAs in humans. To obtain potential therapeutic targetable genes for siRNAs, we used DrugBank 3.0 (www.drugbank.ca) [50]. This database contains curated compound (*e.g.*, drug, chemical) target genes, resulting in 3,540 targets that are considered potential therapeutic targets. Subsequently, intersecting the 3,540 genes from DrugBank and siRNA target genes from the Probe Database, we obtained 25 oncogenic kinases having siRNAs. These genes were inspected throughout TCGA datasets by using cBio Cancer Genomics Portal [44] (detailed in Tables S4–S37 in File S1). Table 2 lists 5 oncogenetic kinases out of 25 total genes.

## Supporting Information

File S1 **The material contains Fig. S1 through S5; and Table S1 through S37.**
(PDF)
