# Supplementary material for: Mg2+ Effect on Argonaute and RNA Duplex by Molecular Dynamics and Bioinformatics Implications
Source: PLoS One. 2014 Oct 17;9(10):e109745. doi: 10.1371/journal.pone.0109745 (PMC4201462; doi:10.1371/journal.pone.0109745)
Supplement: File S1 — The material contains Fig. S1 through S5; and Table S1 through S37. (PDF) [file pone.0109745.s001.pdf]

Supporting Information

Mg^{2+} effect on Argonaute and RNA duplex by molecular dynamics and bioinformatics implications

Seungyoon Nam, Hyojung Ryu, Won-joon Son, Yon Hui Kim, Kyung Tae Kim, Curt Balch, Kenneth P. Nephew, and Jinhyuk Lee

Contents

Supplementary figures S1-S5
Supplementary tables S1-S37
References

Supplementary Figure S1. The molecular dynamics scheme for studying for high Mg^{2+} and low Mg^{2+} concentration Ago/RNA duplex systems. Ago-high contains 10 additional intraceulluar Mg2+ ions while Ago-low contains no additional intraceullular Mg^{2+} ions.

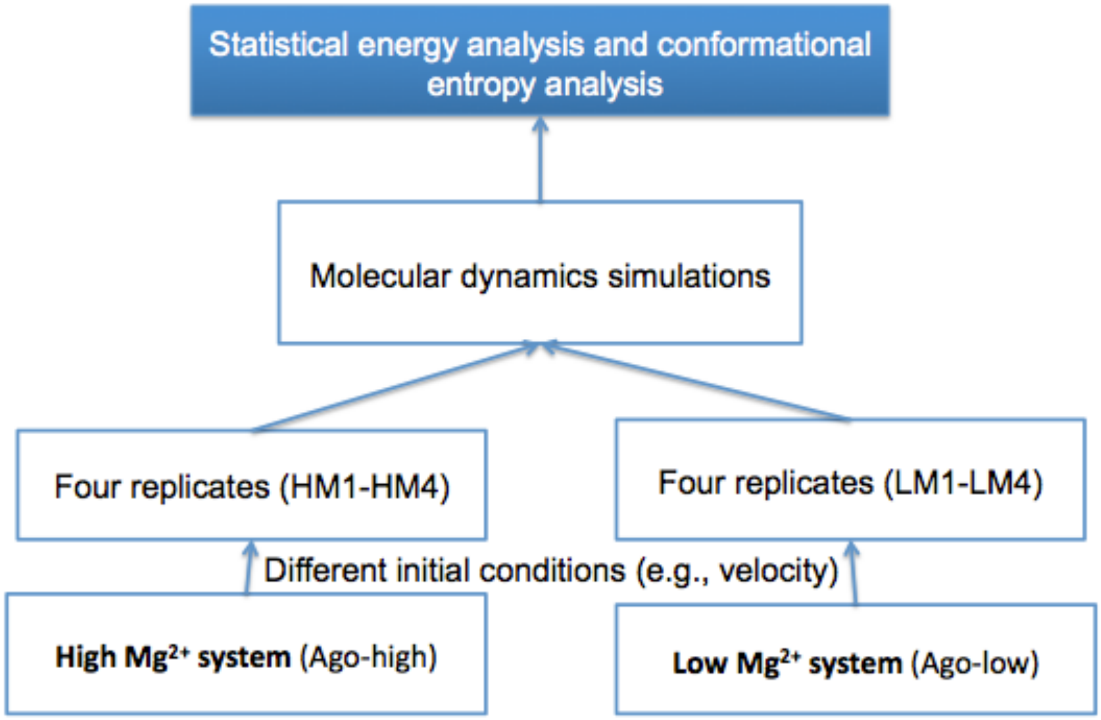

Supplementary Figure S2. Root Mean Square Deviations (RMSDs) of eight systems with respect to the corresponding initial structure in Ago-high (four red lines; HM1-HM4) and Ago-low (four green lines; LM1-LM4). For each system, four replicates were established for molecular dynamics simulation.

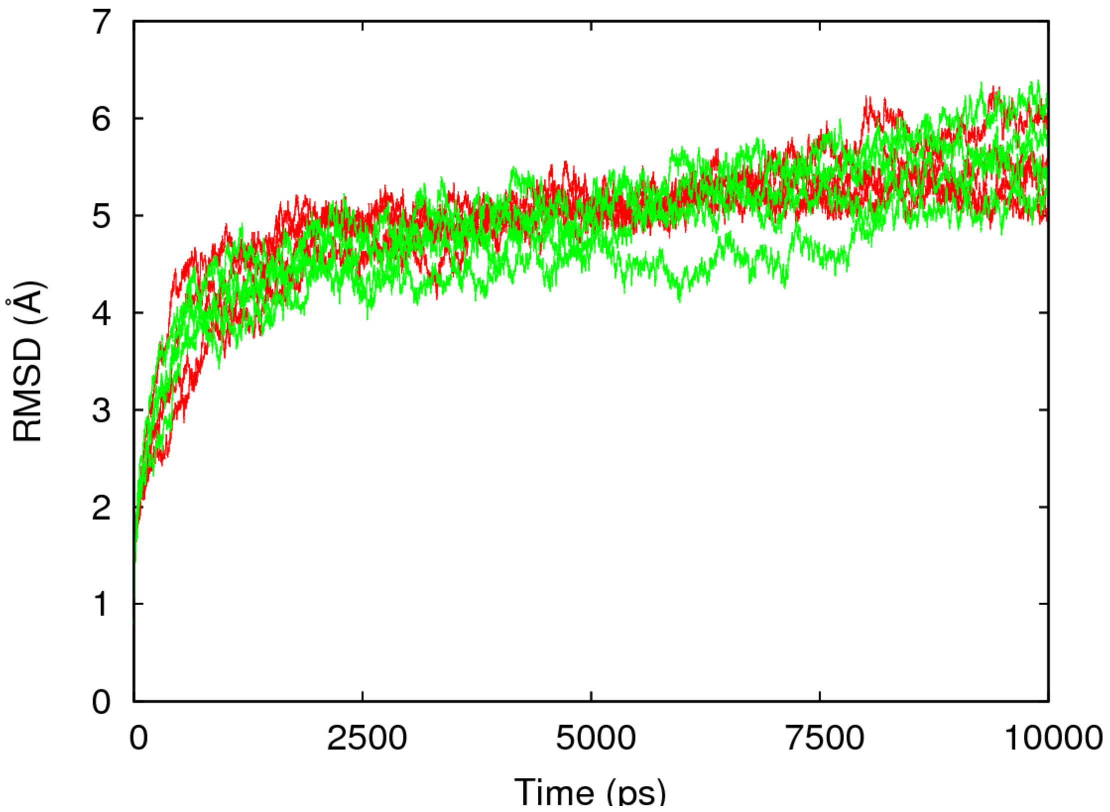

Supplementary Figure S3. **The physical distance of the two PAZ subdomains in Ago-high (red line) and –low (green line) throughout all the simulation time points**. We measured the closest distance from all the pairs between the two subdomains in each time point. The subdomains in Ago-low kept moving closer and away each other in all time points. The distances between two subdomains in Ago-high were not changed during the time course. The same replicates with Fig .1 were used (HM4 for Ago-high and LM2 for Ago-low complex).

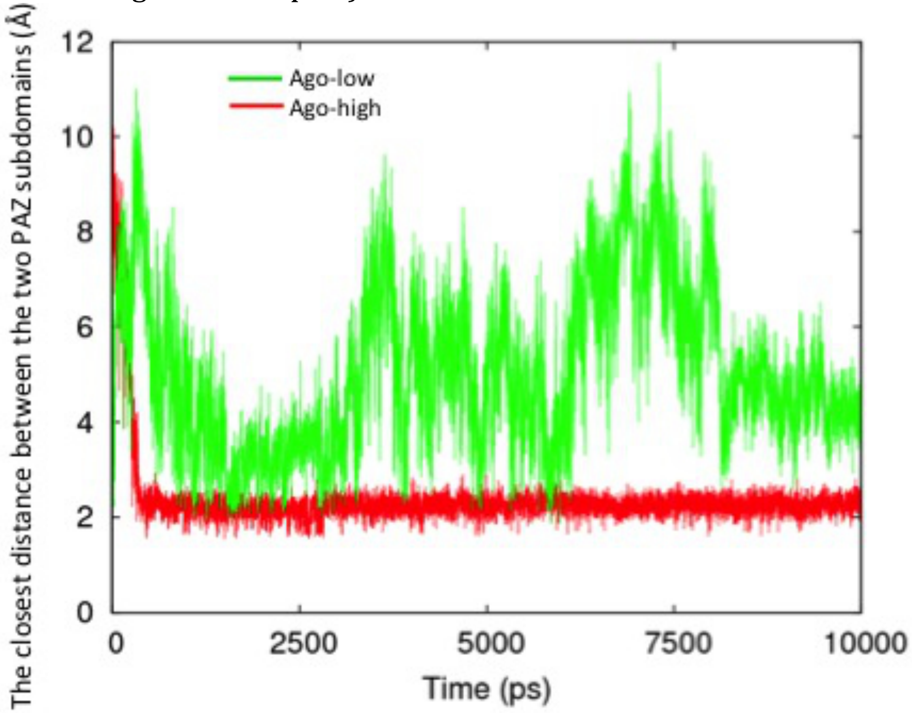

Supplementary Figure S4. The average base-pairing energy between the miRNA and the mRNA (target RNA) as a function of simulation time. (a) In the Ago-high system (high [Mg^{2+}]), average base-pairing energy profiles of the miRNA-target mRNA duplexes were obtained for the four replicates (HM1-4). The interaction energies between two corresponding nucleic acid bases in the duplex are summed and subsequently averaged for the total number of nucleic acids to determine average base-paring energy. (b) In the Ago-low system (low [Mg^{2+}]), the same analysis was done (LM1-4).

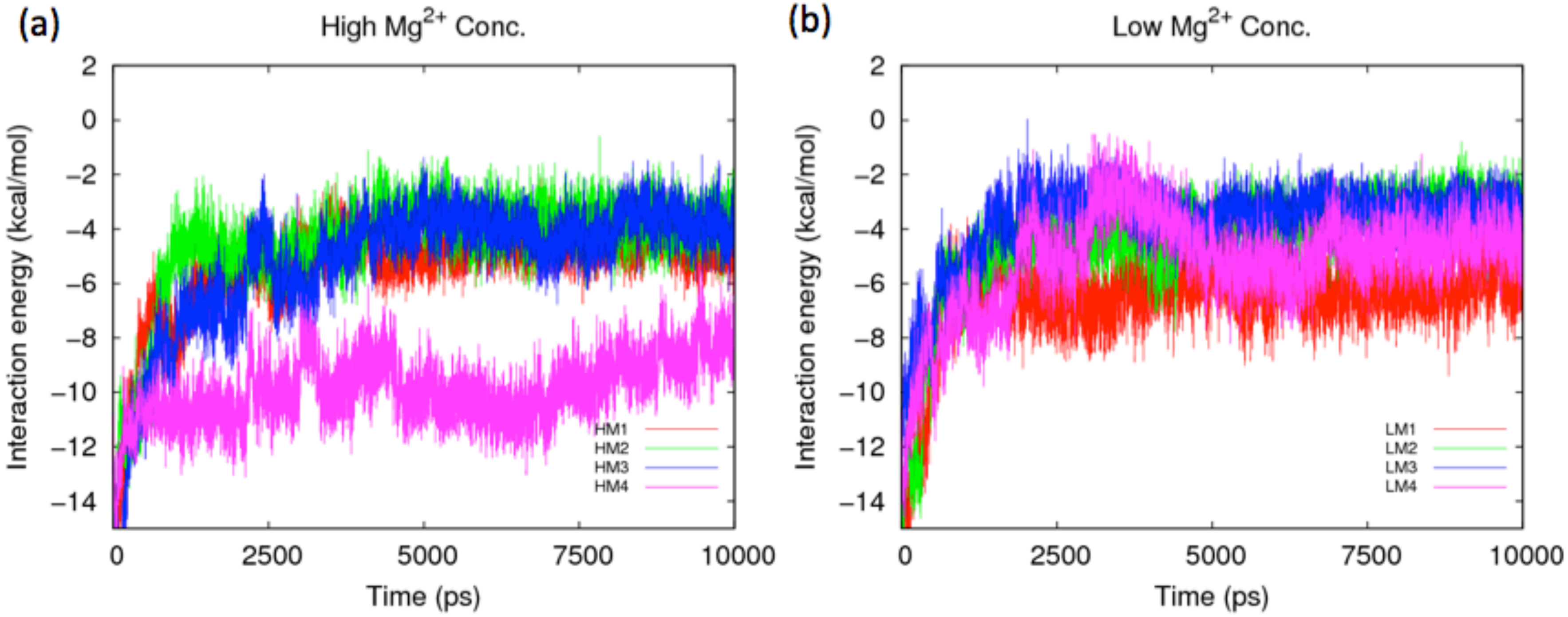

Supplementary Figure S5. Conformational entropy change according to [Mg^{2+}]. The dot represents the average entropy in a given intracellular Mg^{2+} ion concentration. Error bars indicate standard deviations of the entropies. Based on the five average entropies for the five concentrations, correlation was fitted, resulting in R^2 0.738. Thus, the higher the number of the intracellular Mg^{2+} ions, the less flexibility the Ago2 protein.

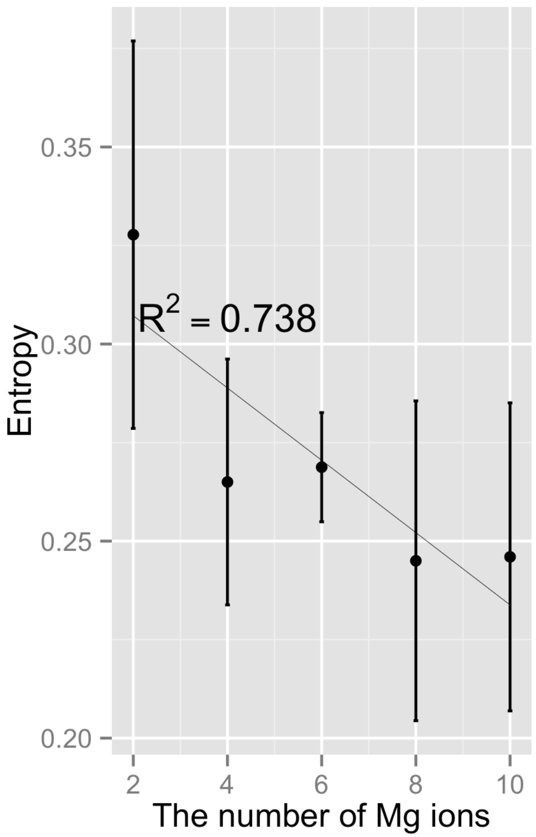

Supplementary Table S1. The key residues bound to the 10 additional Mg^{2+} ions, the inner (PIWI-bound) 2 Mg^{2+} ions, and miRNA-target RNA duplex in Ago-high (*i.e.*, high [Mg^{2+}] condition). The structures used were all final Ago-high structures. The bound key residues were obtained around 5 Å from the three origins (i.e., the 10 additional Mg^{2+} ions, the inner 2 Mg^{2+} ions, and miRNA-target RNA duplex). The negatively charged residues near the additional Mg^{2+} ligands are represented in bold (second row). For the inner 2 Mg^{2+} ions near the catalytic site (the third row), the bold residues are known as catalytic residues involved in slicing the target RNA. The fourth row represents the residues interacting with the RNA duplex.

Origins	Residues within 5 Å from origin
The additional 10 Mg^{2+} ions	PHE10 PHE14 ALA15 **GLU40 GLU41** PRO44 PRO69 PRO70 **GLU71** ARG81 ILE173 SER178 **GLU180 GLU203** LEU205 **GLU209 GLU210 ASP211** LYS213 **GLU214 ASP225** TYR226 LYS230 **GLU237** GLY239 GLY259 THR266 LEU267 **GLU268** TRP283 **GLU284** ARG287 ARG291 **GLU292** SER295 LEU303 PRO306 **GLU307** ALA308 **ASP332** ALA333 **GLU359** PHE360 ARG363 HIS379 HIS382 ALA383 HIS384 ALA390 **GLU393** LYS397 ALA398 **GLU400 GLU401** ALA414 **GLU416 ASP417 GLU441** HIS445 ARG446 **GLU448** LEU452 ALA468 TYR469 **GLU472** GLY495 GLY496 **ASP497** PHE530 ARG536 ALA555 **GLU559** TYR568 VAL584 TYR593 PRO595 GLY612 GLY624 **ASP625 GLU629** GLY646
The inner (PIWI-bound) 2 Mg^{2+} ions in the cleavage site	**ASN478** GLY481 **ASP546** GLY547 LYS575 ARG651 LEU652 **ASP660** VAL663 LYS664
miRNA-target RNA duplex	TYR43 PRO44 ALA47 GLN48 ARG51 ARG59 GLY61 ARG81 ARG114 LEU132 ASP154 PRO169 ALA170 TYR171 ARG172 ILE173 LYS191 ARG192 ARG194 ARG199 THR201 PRO247 VAL264 LEU265 THR266 LEU267 GLU268 LEU281 PRO282 ARG286 LYS329 TRP415 GLN433 ILE434 LEU435 ASN436 HIS445 ARG446 TRP447 ASN449 ALA450 ASN478 ALA479 GLY480 GLY481 GLU483 SER484 ARG486 ALA510 GLY511 GLU512 ARG513 ARG545 ASP546 GLY547 ARG548 VAL549 PRO550 GLN551 VAL573 ARG574 LYS575 SER576 GLY577 ARG580 VAL606 HIS607 ARG611 GLY612 THR613 PRO614 ARG615 LYS618 TYR642 ALA644 SER645 GLY646 PHE647 ALA648 PHE649 PRO650 ARG651 LEU652 HIS657 ASP660 ARG661 LYS664 ARG668 VAL685

Supplementary Table S2. Intermolecular protein-RNA duplex contacts under Ago-low and Ago-high conditions. The contacts between amino acids and nucleic acids were defined using a radius < 2 Å from the duplex phosphate backbones. The middle columns indicate miRNA and target mRNA sequences, respectively. The first two columns represent Mg^{2+} ions and amino acids that have close contacts with the miRNA strand under the two different [Mg^{2+}] (*i.e.*, Ago-high, Ago-low). The last two columns represent Mg^{2+}ions and amino acids that have close contacts with the target mRNA strand under the two different [Mg^{2+}] (*i.e.*, Ago-high, Ago-low). The positions of the inner Mg^{2+} ions having close contacts with the duplex were not changed by the different cation concentrations, unlike the amino acids having contacts with the duplex. Under high [Mg^{2+}] (Ago-high), the additional Mg^{2+} ions were more likely to diffuse to the 5'-end of the miRNA strand.

**Amino acids and Mg^{2+} ions having close contacts with the miRNA**		**RNA duplex**		**Amino acids and Mg^{2+} ions having close contacts with the target mRNA**	
**Ago-high**	**Ago-low**	**miRNA**	**Target mRNA**	**Ago-low**	**Ago-high**
		3'	5'		
		16G	4C	GLY104	SER107, GLY61
GLY131	ARG51	15U	5A	GLN551	GLN551, ARG513
		14C	6G	ARG548, ARG513	GLU512, ARG513
		13A	7U		
ARG574, GLY577	SER576	12G	8C		SER484, LYS248
LYS575, SER576	LYS575, SER576	11A	9U	THR201	THR201
LYS575, Mg(in)[1]	Mg(in)[1]	10C	10G	THR201, ARG200	THR201, ARG199
		9U	11A	TYR135	
	LYS664	8A	12U	TYR171, THR266	
GLU416	GLU416, TRP415	7U	13A	THR613, LEU281, ARG580	TYR135, ARG615, ARG580
	HIS187	6U	14A	ARG651	ARG651
	TRP415	5C	15G	ARG651	ARG651
Mg(add)[2]		4G	16C	TYR642, PRO650	TYR642
Mg(add)[2]		3A	17U	ARG418, Mg(in)[1]	ARG418, Mg(in)[1]
Mg(add)[2]		2U	18A		
		5'	3'		

[1]Mg(in) indicates the PIWI-bound Mg^{2+} ion inside Ago2 protein.
[2]Mg(add) indicates the additional Mg^{2+} ion outside Ago2 protein in Ago-high.

Supplementary Table S3. Ago-related crystallography structures and their characteristics in the RCSB Protein Data Bank. Out of the two structures (3HK2, 3HVR) having the longest duplexes, 3HK2 had better resolution than 3HVR, which led to our selection of 3HK2 as a base ternary structure in our study.

PDB ID	Species	Duplex availability	Duplex length	Resolution (Å)	Publication
3HK2	Thermus thermophilus	Yes	RNA: 19 nt; DNA: 21 nt	2.80	[1]
4OLA	Homo sapiens	No (only single strand)	RNA: 10 nt	2.30	[2]
4OLB	Homo sapiens	No (only single strand)	RNA: 10 nt	2.90	[2]
3HVR	Thermus thermophilus	Yes	RNA: 19 nt; DNA: 21 nt	3.21	[1]
3HO1	Thermus thermophilus	Yes	RNA: 12 nt; DNA: 21 nt	2.60	[1]

Supplementary Table S4. Implication of potential effects of Mg^{2+} ions on RNAi in basal subtype breast cancer. All the 25 oncogenic kinases are listed. We set "Select Cancer Study", "Select Genomic Profiles", "Enter a z-score threshold", and "Select Patient/Case Set" to "Breast Invasive Carcinoma (TCGA, Nature 2012)", "mRNA Expression z-Scores (microarray)", 1.0, and "PAM50 Basal", respectively, in cBioportal.org. The z-score threshold is used to identify highly or lowly regulated genes. According to high or low *TRPM7* expression, we divided the basal subtype breast cancer patients (total 49 patients). The numeric in a cell represents the proportion of patients with high expression of the given gene.

**25 oncogenic kinases**	***TRPM7* up-regulated patients (12 pts) : Mg^{2+} high patients (%)**	***TRPM7* down-regulated patients (37 pts) : Mg^{2+} low patients (%)**
ABL1	8.33<br>(% of the 12 patients have high ABL1 expression)	16.22<br>(% of the 37 patients have high ABL1 expression)
ABL2	41.67	62.16
AKT1	0	10.81
AKT2	8.33	29.73
BRAF	50	43.24
CDK4	25	48.65
CDK6	83.33	54.05
EGFR	75	91.89
ERBB2	0	2.7
FGFR1	16.67	21.62
FGFR2	25	24.32
FGFR3	16.67	8.11
FLT3	8.33	2.7
ITK	41.67	13.51
KDR	0	8.11
KIT	41.67	48.65
LCK	25	18.92
MET	75	54.05
NTRK1	0	0
PDGFRA	16.67	5.41
PDGFRB	8.33	5.41
PIM1	75	43.24
RAF1	33.33	27.02
RET	8.33	0
SYK	41.67	29.73

Supplementary Table S5. Implication of potential effects of Mg^{2+} ions on RNAi in Claudin-low subtype breast cancer. We set "Select Cancer Study", "Select Genomic Profiles", "Enter a z-score threshold", and "Select Patient/Case Set" to "Breast Invasive Carcinoma (TCGA, Nature 2012)", "mRNA Expression z-Scores (microarray)", 1.0, and "PAM50 Claudin low", respectively, in cBioportal.org. According to high or low *TRPM7* expression, we divided the subtype breast cancer patients (total 4 patients). The numeric in a cell represents the proportion of patients with high expression of the given gene.

**25 oncogenic kinases**	***TRPM7* up-regulated patients (2 pts) : Mg^{2+} high patients (%)**	***TRPM7* down-regulated patients (2 pts) : Mg^{2+} low patients (%)**
ABL1	0	50
ABL2	100	100
AKT1	0	0
AKT2	0	0
BRAF	0	50
CDK4	0	0
CDK6	0	50
EGFR	0	50
ERBB2	0	0
FGFR1	0	100
FGFR2	0	50
FGFR3	0	0
FLT3	0	0
ITK	100	0
KDR	50	0
KIT	50	0
LCK	100	0
MET	50	50
NTRK1	0	0
PDGFRA	100	50
PDGFRB	50	100
PIM1	100	50
RAF1	0	100
RET	0	0
SYK	50	50

Supplementary Table S6. Implication of potential effects of Mg^{2+} ions on RNAi in Her2-enriched subtype breast cancer. We set "Select Cancer Study", "Select Genomic Profiles", "Enter a z-score threshold", and "Select Patient/Case Set" to "Breast Invasive Carcinoma (TCGA, Nature 2012)", "mRNA Expression z-Scores (microarray)", 1.0, and "Her2 enriched", respectively, in cBioportal.org. According to high or low *TRPM7* expression, we divided the subtype breast cancer patients (total 25 patients).

**25 oncogenic kinases**	***TRPM7* up-regulated patients (5 pts) : Mg^{2+} high patients (%)**	***TRPM7* down-regulated patients (20 pts) : Mg^{2+} low patients (%)**
ABL1	0	45
ABL2	40	30
AKT1	20	50
AKT2	20	15
BRAF	0	5
CDK4	0	35
CDK6	0	15
EGFR	40	30
ERBB2	80	75
FGFR1	20	15
FGFR2	20	5
FGFR3	0	5
FLT3	20	5
ITK	0	15
KDR	60	30
KIT	20	5
LCK	0	20
MET	20	25
NTRK1	0	10
PDGFRA	0	5
PDGFRB	0	15
PIM1	0	5
RAF1	20	35
RET	40	15
SYK	20	15

Supplementary Table S7. Implication of potential effects of Mg^{2+} ions on RNAi in Luminal A subtype breast cancer. We set "Select Cancer Study", "Select Genomic Profiles", "Enter a z-score threshold", and "Select Patient/Case Set" to "Breast Invasive Carcinoma (TCGA, Nature 2012)", "mRNA Expression z-Scores (microarray)", 1.0, and "PAM50 Luminal A", respectively, in cBioportal.org. According to high or low *TRPM7* expression, we divided the subtype breast cancer patients (total 69 patients).

**25 oncogenic kinases**	***TRPM7* up-regulated patients (36 pts) : Mg^{2+} high patients (%)**	***TRPM7* down-regulated patients (33 pts) : Mg^{2+} low patients (%)**
ABL1	8.33	30.3
ABL2	36.11	36.36
AKT1	11.11	24.24
AKT2	27.78	18.18
BRAF	27.78	15.15
CDK4	8.33	3.03
CDK6	0	6.06
EGFR	5.56	3.03
ERBB2	36.11	18.18
FGFR1	30.56	24.24
FGFR2	16.67	15.15
FGFR3	27.78	3.03
FLT3	36.11	15.15
ITK	8.33	6.06
KDR	22.22	6.06
KIT	2.78	9.09
LCK	8.33	6.06
MET	8.33	9.09
NTRK1	16.67	9.09
PDGFRA	19.44	18.18
PDGFRB	13.89	33.33
PIM1	8.33	9.09
RAF1	16.67	15.15
RET	19.44	12.12
SYK	8.33	3.03

Supplementary Table S8. Implication of potential effects of Mg^{2+} ions on RNAi in Luminal B subtype breast cancer. We set "Select Cancer Study", "Select Genomic Profiles", "Enter a z-score threshold", and "Select Patient/Case Set" to "Breast Invasive Carcinoma (TCGA, Nature 2012)", "mRNA Expression z-Scores (microarray)", 1.0, and "PAM50 Luminal B", respectively, in cBioportal.org. According to high or low *TRPM7* expression, we divided the subtype breast cancer patients (total 49 patients).

**25 oncogenic kinases**	***TRPM7* up-regulated patients (24 pts) : Mg^{2+} high patients (%)**	***TRPM7* down-regulated patients (25 pts) : Mg^{2+} low patients (%)**
ABL1	8.33	16
ABL2	20.83	12
AKT1	4.17	28
AKT2	41.67	20
BRAF	20.83	8
CDK4	29.17	32
CDK6	0	4
EGFR	4.17	4
ERBB2	25	32
FGFR1	37.5	16
FGFR2	12.5	12
FGFR3	50	32
FLT3	37.5	8
ITK	8.33	8
KDR	4.17	8
KIT	0	0
LCK	8.33	8
MET	4.17	4
NTRK1	20.83	12
PDGFRA	0	4
PDGFRB	4.17	16
PIM1	8.33	4
RAF1	25	36
RET	41.67	28
SYK	4.17	0

Supplementary Table S9. Implication of potential effects of Mg^{2+} ions on RNAi in acute myeloid leukemia. We set "Select Cancer Study", "Select Genomic Profiles", "Enter a z-score threshold", and "Select Patient/Case Set" to "Acute Myeloid Leukemia (TCGA, NEJM 2013)", "mRNA Expression z-Scores (RNA Seq V2 RSEM)", 1.0, and "Tumors with mRNA data (RNA Seq V2)", respectively, in cBioportal.org. According to high or low *TRPM7* expression, we divided the cancer patients (total 44 patients).

**25 oncogenic kinases**	***TRPM7* up-regulated patients (21 pts) : Mg^{2+} high patients (%)**	***TRPM7* down-regulated patients (23 pts) : Mg^{2+} low patients (%)**
ABL1	14.29	30.43
ABL2	47.62	0
AKT1	4.76	52.17
AKT2	0	34.78
BRAF	42.86	0
CDK4	0	39.13
CDK6	38.1	4.35
EGFR	14.29	4.35
ERBB2	9.52	13.04
FGFR1	4.76	8.7
FGFR2	4.76	0
FGFR3	4.76	8.7
FLT3	23.81	4.35
ITK	0	8.7
KDR	9.52	4.35
KIT	9.52	4.35
LCK	0	13.04
MET	14.29	0
NTRK1	4.76	0
PDGFRA	19.05	0
PDGFRB	19.05	13.04
PIM1	14.29	17.39
RAF1	0	4.35
RET	0	13.04
SYK	9.52	26.09

Supplementary Table S10. Implication of potential effects of Mg^{2+} ions on RNAi in bladder cancer. We set "Select Cancer Study", "Select Genomic Profiles", "Enter a z-score threshold", and "Select Patient/Case Set" to "Bladder Cancer (MSKCC, JCO 2013)", "mRNA Expression z-Scores (Illumina)", 1.0, and "Tumors with mRNA data (Agilent microarray)", respectively, in cBioportal.org. According to high or low *TRPM7* expression, we divided the cancer patients (total 20 patients).

**25 oncogenic kinases**	***TRPM7* up-regulated patients (11 pts) : Mg^{2+} high patients (%)**	***TRPM7* down-regulated patients (9 pts) : Mg^{2+} low patients (%)**
ABL1	9.09	0
ABL2	18.18	11.11
AKT1	9.09	0
AKT2	18.18	11.11
BRAF	36.36	22.22
CDK4	18.18	22.22
CDK6	18.18	11.11
EGFR	18.18	22.22
ERBB2	27.27	0
FGFR1	9.09	11.11
FGFR2	9.09	33.33
FGFR3	9.09	22.22
FLT3	9.09	11.11
ITK	27.27	11.11
KDR	0	22.22
KIT	18.18	33.33
LCK	27.27	11.11
MET	27.27	22.22
NTRK1	9.09	11.11
PDGFRA	18.18	22.22
PDGFRB	0	0
PIM1	18.18	33.33
RAF1	18.18	11.11
RET	9.09	11.11
SYK	9.09	0

Supplementary Table S11. Implication of potential effects of Mg^{2+} ions on RNAi in bladder uroepithelial Carcinoma. We set "Select Cancer Study", "Select Genomic Profiles", "Enter a z-score threshold", and "Select Patient/Case Set" to "Bladder Uroepithelial Carcinoma (TCGA, provisional)", "mRNA Expression z-Scores (RNA Seq V2 RSEM)", 1.0, and "Tumors with mRNA data (RNA Seq V2)", respectively, in cBioportal.org. According to high or low *TRPM7* expression, we divided the cancer patients (total 70 patients).

**25 oncogenic kinases**	***TRPM7* up-regulated patients (30 pts) : Mg^{2+} high patients (%)**	***TRPM7* down-regulated patients (40 pts) : Mg^{2+} low patients (%)**
ABL1	10	7.5
ABL2	16.67	12.5
AKT1	10	17.5
AKT2	23.33	40
BRAF	26.67	12.5
CDK4	16.67	25
CDK6	16.67	17.5
EGFR	20	22.5
ERBB2	26.67	12.5
FGFR1	20	7.5
FGFR2	30	10
FGFR3	10	5
FLT3	10	7.5
ITK	3.33	0
KDR	6.67	5
KIT	3.33	10
LCK	3.33	2.5
MET	10	22.5
NTRK1	0	5
PDGFRA	16.67	5
PDGFRB	13.33	7.5
PIM1	13.33	15
RAF1	50	40
RET	3.33	2.5
SYK	10	2.5

Supplementary Table S12. Implication of potential effects of Mg^{2+} ions on RNAi in brain lower grade glioma. We set "Select Cancer Study", "Select Genomic Profiles", "Enter a z-score threshold", and "Select Patient/Case Set" to "Brain Lower Grade Glioma (TCGA, provisional)", "mRNA Expression z-Scores (RNA Seq V2 RSEM)", 1.0, and "Tumors with mRNA data (RNA Seq V2)", respectively, in cBioportal.org. According to high or low *TRPM7* expression, we divided the cancer patients (total 44 patients).

**25 oncogenic kinases**	***TRPM7* up-regulated patients (21 pts) : Mg^{2+} high patients (%)**	***TRPM7* down-regulated patients (23 pts) : Mg^{2+} low patients (%)**
ABL1	23.81	0
ABL2	23.81	8.7
AKT1	9.52	17.39
AKT2	23.81	8.7
BRAF	28.57	0
CDK4	4.76	26.09
CDK6	23.81	0
EGFR	42.86	0
ERBB2	33.33	0
FGFR1	33.33	8.7
FGFR2	0	8.7
FGFR3	19.05	4.35
FLT3	23.81	8.7
ITK	28.57	0
KDR	14.29	0
KIT	0	13.04
LCK	38.1	0
MET	19.05	17.39
NTRK1	9.52	4.35
PDGFRA	14.29	8.7
PDGFRB	14.29	0
PIM1	19.05	13.04
RAF1	28.57	4.35
RET	0	17.39
SYK	38.1	8.7

Supplementary Table S13. Implication of potential effects of Mg^{2+} ions on RNAi in breast cancer. We set "Select Cancer Study", "Select Genomic Profiles", "Enter a z-score threshold", and "Select Patient/Case Set" to "Breast Invasive Carcinoma (TCGA, provisional)", "mRNA Expression z-Scores (RNA Seq V2 RSEM)", 1.0, and "Tumors with mRNA data (RNA Seq V2)", respectively, in cBioportal.org. According to high or low *TRPM7* expression, we divided the cancer patients (total 367 patients).

**25 oncogenic kinases**	***TRPM7* up-regulated patients (133 pts) : Mg^{2+} high patients (%)**	***TRPM7* down-regulated patients (234 pts) : Mg^{2+} low patients (%)**
ABL1	12.03	15.38
ABL2	46.62	17.09
AKT1	11.28	23.93
AKT2	16.54	23.93
BRAF	27.82	8.97
CDK4	5.26	30.34
CDK6	10.53	13.68
EGFR	10.53	10.26
ERBB2	27.82	23.93
FGFR1	15.79	12.39
FGFR2	12.78	13.25
FGFR3	9.77	5.98
FLT3	15.04	5.98
ITK	9.77	5.56
KDR	15.79	8.12
KIT	5.26	7.69
LCK	6.02	8.97
MET	14.29	13.25
NTRK1	0.75	0.43
PDGFRA	16.54	5.13
PDGFRB	8.27	10.68
PIM1	3.76	15.81
RAF1	18.05	23.08
RET	12.03	5.98
SYK	17.29	11.11

Supplementary Table S14. Implication of potential effects of Mg^{2+} ions on RNAi in cervical cancer. We set “Select Cancer Study”, “Select Genomic Profiles”, “Enter a z-score threshold”, and “Select Patient/Case Set” to “Cervical Squamous Cell Carcinoma and Endocervical Adenocarcinoma (TCGA, Provisional)”, “mRNA Expression z-Scores (RNA Seq V2 RSEM)”, 1.0, and “Tumors with mRNA data (RNA Seq V2)”, respectively, in cBioportal.org. According to high or low *TRPM7* expression, we divided the cancer patients (total 46 patients).

**25 oncogenic kinases**	***TRPM7* up-regulated patients (19 pts) : Mg^{2+} high patients (%)**	***TRPM7* down-regulated patients (27 pts) : Mg^{2+} low patients (%)**
ABL1	26.32	18.52
ABL2	31.58	37.04
AKT1	31.58	33.33
AKT2	52.63	40.74
BRAF	36.84	18.52
CDK4	21.05	22.22
CDK6	5.26	25.93
EGFR	10.53	18.52
ERBB2	26.32	18.52
FGFR1	5.26	3.7
FGFR2	15.79	3.7
FGFR3	10.53	7.41
FLT3	10.53	0
ITK	5.26	0
KDR	21.05	14.81
KIT	5.26	3.7
LCK	5.26	11.11
MET	10.53	22.22
NTRK1	10.53	25.93
PDGFRA	10.53	7.41
PDGFRB	21.05	14.81
PIM1	26.32	18.52
RAF1	5.26	7.41
RET	10.53	7.41
SYK	10.53	0

Supplementary Table S15. Implication of potential effects of Mg^{2+} ions on RNAi in colorectal cancer (1). We set “Select Cancer Study”, “Select Genomic Profiles”, “Enter a z-score threshold”, and “Select Patient/Case Set” to “Colon and Rectum Adenocarcinoma (TCGA, Nature 2012)”, “mRNA Expression z-Scores (RNA Seq RPKM)”, 1.0, and “Tumors with mRNA data (RNA Seq)”, respectively, in cBioportal.org. According to high or low *TRPM7* expression, we divided the cancer patients (total 63 patients).

**25 oncogenic kinases**	***TRPM7* up-regulated patients (22 pts) : Mg^{2+} high patients (%)**	***TRPM7* down-regulated patients (41 pts) : Mg^{2+} low patients (%)**
ABL1	13.64	14.63
ABL2	50	12.2
AKT1	0	43.9
AKT2	4.55	56.1
BRAF	40.91	0
CDK4	13.64	12.2
CDK6	54.55	4.88
EGFR	27.27	21.95
ERBB2	13.64	39.02
FGFR1	13.64	29.27
FGFR2	40.91	4.88
FGFR3	18.18	14.63
FLT3	13.64	2.44
ITK	4.55	0
KDR	13.64	0
KIT	9.09	0
LCK	0	2.44
MET	27.27	9.76
NTRK1	0	31.71
PDGFRA	22.73	4.88
PDGFRB	4.55	26.83
PIM1	9.09	24.39
RAF1	40.91	4.88
RET	13.64	12.2
SYK	18.18	7.32

Supplementary Table S16. Implication of potential effects of Mg^{2+} ions on RNAi in colorectal cancer (2). We set "Select Cancer Study", "Select Genomic Profiles", "Enter a z-score threshold", and "Select Patient/Case Set" to "Colon and Rectum Adenocarcinoma (TCGA, Provisional)", "mRNA Expression z-Scores (RNA Seq RPKM)", 1.0, and "Tumors with mRNA data (RNA Seq)", respectively, in cBioportal.org. Subsequently, we used MSI status information according to TRPM7 up- or down-regulated expression of the patients via "Build a Case Set".

**Histology**	**MSI-H**		**MSS**		**MSI-L**		**Not evaluable**	
**25 oncogenic kinases**	*TRPM7* **up-regulated patients (0 pts) : Mg^{2+} high patients (%)**	*TRPM7* **down-regulated patients (1 pts) : Mg^{2+} low patients (%)**	*TRPM7* **up-regulated patients (0 pts) : Mg^{2+} high patients (%)**	*TRPM7* **down-regulated patients (3 pts) : Mg^{2+} low patients (%)**	*TRPM7* **up-regulated patients (0 pts) : Mg^{2+} high patients (%)**	*TRPM7* **down-regulated patients (2 pts) : Mg^{2+} low patients (%)**	*TRPM7* **up-regulated patients (0 pts) : Mg^{2+} high patients (%)**	*TRPM7* **down-regulated patients (0 pts) : Mg^{2+} low patients (%)**
ABL1	0	0	0	0	0	0	0	0
ABL2	0	0	0	33.33	0	0	0	0
AKT1	0	100	0	0	0	50	0	0
AKT2	0	0	0	0	0	50	0	0
BRAF	0	0	0	0	0	0	0	0
CDK4	0	0	0	33.33	0	0	0	0
CDK6	0	100	0	0	0	0	0	0
EGFR	0	0	0	33.33	0	50	0	0
ERBB2	0	0	0	33.33	0	0	0	0
FGFR1	0	0	0	33.33	0	0	0	0
FGFR2	0	0	0	0	0	0	0	0
FGFR3	0	0	0	33.33	0	0	0	0
FLT3	0	0	0	0	0	50	0	0

ITK	0	0	0	0	0	0	0	0
KDR	0	0	0	0	0	0	0	0
KIT	0	0	0	33.33	0	0	0	0
LCK	0	100	0	0	0	0	0	0
MET	0	0	0	33.33	0	100	0	0
NTRK1	0	0	0	0	0	0	0	0
PDGFRA	0	0	0	0	0	0	0	0
PDGFRB	0	0	0	0	0	0	0	0
PIM1	0	0	0	0	0	50	0	0
RAF1	0	0	0	0	0	0	0	0
RET	0	0	0	0	0	50	0	0
SYK	0	0	0	33.33	0	0	0	0

Supplementary Table S17. Implication of potential effects of Mg^{2+} ions on RNAi in glioblastoma (1). We set "Select Cancer Study", "Select Genomic Profiles", "Enter a z-score threshold", and "Select Patient/Case Set" to "Glioblastoma (TCGA, Nature 2008)", "mRNA Expression z-Scores (microarray)", 1.0, and "All Tumors", respectively, in cBioportal.org. According to high or low *TRPM7* expression, we divided the cancer patients (total 5 patients).

**25 oncogenic kinases**	***TRPM7* up-regulated patients (1 pts) : Mg^{2+} high patients (%)**	***TRPM7* down-regulated patients (4 pts) : Mg^{2+} low patients (%)**
ABL1	0	0
ABL2	0	0
AKT1	0	0
AKT2	0	0
BRAF	0	0
CDK4	0	50
CDK6	0	0
EGFR	0	0
ERBB2	0	0
FGFR1	0	0
FGFR2	0	0
FGFR3	0	0
FLT3	0	0
ITK	0	0
KDR	0	25
KIT	0	0
LCK	0	0
MET	0	0
NTRK1	0	0
PDGFRA	0	0
PDGFRB	0	0
PIM1	0	0
RAF1	0	0
RET	0	0
SYK	0	25

Supplementary Table S18. Implication of potential effects of Mg^{2+} ions on RNAi in glioblastoma (2). We set "Select Cancer Study", "Select Genomic Profiles", "Enter a z-score threshold", and "Select Patient/Case Set" to "Glioblastoma Multiforme (TCGA, Provisional)", "mRNA Expression z-Scores (RNA Seq V2 RSEM)", 1.0, and "Tumors with mRNA data (RNA Seq V2)", respectively, in cBioportal.org. According to high or low *TRPM7* expression, we divided the cancer patients (total 53 patients).

**25 oncogenic kinases**	***TRPM7* up-regulated patients (20 pts) : Mg^{2+} high patients (%)**	***TRPM7* down-regulated patients (33 pts) : Mg^{2+} low patients (%)**
ABL1	20	18.18
ABL2	25	3.03
AKT1	0	0
AKT2	5	21.21
BRAF	55	18.18
CDK4	40	60.61
CDK6	30	6.06
EGFR	35	36.36
ERBB2	15	3.03
FGFR1	30	6.06
FGFR2	10	18.18
FGFR3	0	0
FLT3	20	6.06
ITK	10	3.03
KDR	20	3.03
KIT	10	33.33
LCK	10	6.06
MET	40	24.24
NTRK1	25	9.09
PDGFRA	25	33.33
PDGFRB	10	9.09
PIM1	5	9.09
RAF1	20	36.36
RET	0	0
SYK	10	3.03

Supplementary Table S19. Implication of potential effects of Mg^{2+} ions on RNAi in head and neck squamous cell carcinoma (1). We set "Select Cancer Study", "Select Genomic Profiles", "Enter a z-score threshold", and "Select Patient/Case Set" to "Head and Neck Squamous Cell Carcinoma (TCGA, In preparation)", "mRNA Expression z-Scores (RNA Seq V2 RSEM)", 1.0, and "Tumors with mRNA data (RNA Seq V2)", respectively, in cBioportal.org. According to high or low *TRPM7* expression, we divided the cancer patients (total 59 patients).

**25 oncogenic kinases**	***TRPM7* up-regulated patients (30 pts) : Mg^{2+} high patients (%)**	***TRPM7* down-regulated patients (29 pts) : Mg^{2+} low patients (%)**
ABL1	20.00	13.79
ABL2	3.33	17.24
AKT1	23.33	27.59
AKT2	13.33	34.48
BRAF	46.67	10.34
CDK4	33.33	13.79
CDK6	16.67	17.24
EGFR	33.33	24.14
ERBB2	20.00	17.24
FGFR1	20.00	6.90
FGFR2	40.00	0.00
FGFR3	13.33	3.45
FLT3	16.67	0.00
ITK	16.67	0.00
KDR	6.67	0.00
KIT	10.00	0.00
LCK	10.00	3.45
MET	10.00	13.79
NTRK1	3.33	0.00
PDGFRA	16.67	0.00
PDGFRB	0.00	13.79
PIM1	0.00	13.79
RAF1	13.33	6.90
RET	10.00	10.34
SYK	40.00	10.34

Supplementary Table S20. Implication of potential effects of Mg^{2+} ions on RNAi in head and neck squamous cell carcinoma (2). We set "Select Cancer Study", "Select Genomic Profiles", "Enter a z-score threshold", and "Select Patient/Case Set" to "Head and Neck Squamous Cell Carcinoma (TCGA, Provisional)", "mRNA Expression z-Scores (RNA Seq V2 RSEM)", 1.0, and "Tumors with mRNA data (RNA Seq V2)", respectively, in cBioportal.org. According to high or low *TRPM7* expression, we divided the cancer patients (total 64 patients).

**25 oncogenic kinases**	***TRPM7* up-regulated patients (34 pts) : Mg^{2+} high patients (%)**	***TRPM7* down-regulated patients (30 pts) : Mg^{2+} low patients (%)**
ABL1	20.59	13.33
ABL2	2.94	16.67
AKT1	23.53	26.67
AKT2	11.76	33.33
BRAF	47.06	10
CDK4	32.35	13.33
CDK6	14.71	16.67
EGFR	29.41	23.33
ERBB2	20.59	16.67
FGFR1	23.53	6.67
FGFR2	41.18	0
FGFR3	11.76	3.33
FLT3	20.59	0
ITK	20.59	0
KDR	5.88	0
KIT	8.82	0
LCK	14.71	3.33
MET	8.82	13.33
NTRK1	2.94	0
PDGFRA	14.71	0
PDGFRB	0	13.33
PIM1	0	13.33
RAF1	11.76	6.67
RET	8.82	10
SYK	38.24	10

Supplementary Table S21. Implication of potential effects of Mg^{2+} ions on RNAi in kidney chromophobe. We set “Select Cancer Study”, “Select Genomic Profiles”, “Enter a z-score threshold”, and “Select Patient/Case Set” to “Kidney Chromophobe (TCGA, Provisional)”, “mRNA Expression z-Scores (RNA Seq V2 RSEM)”, 1.0, and “Tumors with mRNA data (RNA Seq V2)”, respectively, in cBioportal.org. According to high or low *TRPM7* expression, we divided the cancer patients (total 17 patients).

**25 oncogenic kinases**	***TRPM7* up-regulated patients (8 pts) : Mg^{2+} high patients (%)**	***TRPM7* down-regulated patients (9 pts) : Mg^{2+} low patients (%)**
ABL1	12.5	33.33
ABL2	0	11.11
AKT1	0	55.56
AKT2	25	44.44
BRAF	25	0
CDK4	25	55.56
CDK6	37.5	11.11
EGFR	12.5	11.11
ERBB2	0	22.22
FGFR1	0	0
FGFR2	12.5	33.33
FGFR3	0	11.11
FLT3	12.5	0
ITK	0	0
KDR	0	11.11
KIT	0	0
LCK	0	0
MET	25	0
NTRK1	0	22.22
PDGFRA	0	11.11
PDGFRB	0	11.11
PIM1	0	0
RAF1	25	44.44
RET	0	11.11
SYK	0	11.11

Supplementary Table S22. Implication of potential effects of Mg^{2+} ions on RNAi in kidney renal clear cell carcinoma (1). We set “Select Cancer Study”, “Select Genomic Profiles”, “Enter a z-score threshold”, and “Select Patient/Case Set” to “Kidney Renal Clear Cell Carcinoma (TCGA, in press)”, “mRNA Expression z-Scores (RNA Seq V2 RSEM)”, 1.0, and “Tumors with mRNA data (RNA Seq V2)”, respectively, in cBioportal.org. According to high or low *TRPM7* expression, we divided the cancer patients (total 107 patients).

**25 oncogenic kinases**	***TRPM7* up-regulated patients (57 pts) : Mg^{2+} high patients (%)**	***TRPM7* down-regulated patients (50 pts) : Mg^{2+} low patients (%)**
ABL1	8.77	20
ABL2	10.53	20
AKT1	8.77	18
AKT2	14.04	28
BRAF	50.88	6
CDK4	14.04	42
CDK6	31.58	12
EGFR	22.81	2
ERBB2	26.32	10
FGFR1	12.28	22
FGFR2	22.81	10
FGFR3	8.77	4
FLT3	1.75	2
ITK	3.51	6
KDR	12.28	4
KIT	12.28	6
LCK	0	24
MET	29.82	28
NTRK1	3.51	12
PDGFRA	8.77	16
PDGFRB	1.75	28
PIM1	17.54	22
RAF1	14.04	6
RET	0	8
SYK	14.04	12

Supplementary Table S23. Implication of potential effects of Mg^{2+} ions on RNAi in kidney renal clear cell carcinoma (2). We set "Select Cancer Study", "Select Genomic Profiles", "Enter a z-score threshold", and "Select Patient/Case Set" to "Kidney Renal Clear Cell Carcinoma (TCGA, provisional)", "mRNA Expression z-Scores (RNA Seq V2 RSEM)", 1.0, and "Tumors with mRNA data (RNA Seq V2)", respectively, in cBioportal.org. According to high or low *TRPM7* expression, we divided the cancer patients (total 112 patients).

**25 oncogenic kinases**	***TRPM7* up-regulated patients (59 pts) : Mg^{2+} high patients (%)**	***TRPM7* down-regulated patients (53 pts) : Mg^{2+} low patients (%)**
ABL1	8.47	20.75
ABL2	10.17	18.87
AKT1	8.47	16.98
AKT2	13.56	26.42
BRAF	52.54	5.66
CDK4	13.56	41.51
CDK6	30.51	11.32
EGFR	23.73	1.89
ERBB2	28.81	11.32
FGFR1	13.56	24.53
FGFR2	23.73	11.32
FGFR3	8.47	3.77
FLT3	1.69	1.89
ITK	3.39	5.66
KDR	11.86	1.89
KIT	15.25	5.66
LCK	0	22.64
MET	28.81	26.42
NTRK1	3.39	11.32
PDGFRA	10.17	15.09
PDGFRB	1.69	24.53
PIM1	18.64	20.75
RAF1	15.25	9.43
RET	0	5.66
SYK	13.56	11.32

Supplementary Table S24. Implication of potential effects of Mg^{2+} ions on RNAi in kidney renal papillary cell carcinoma. We set "Select Cancer Study", "Select Genomic Profiles", "Enter a z-score threshold", and "Select Patient/Case Set" to "Kidney Renal Papillary Cell Carcinoma (TCGA, provisional)", "mRNA Expression z-Scores (RNA Seq V2 RSEM)", 1.0, and "Tumors with mRNA data (RNA Seq V2)", respectively, in cBioportal.org. According to high or low *TRPM7* expression, we divided the cancer patients (total 8 patients)

**25 oncogenic kinases**	***TRPM7* up-regulated patients (6 pts) : Mg^{2+} high patients (%)**	***TRPM7* down-regulated patients (2 pts) : Mg^{2+} low patients (%)**
ABL1	66.67	0
ABL2	0	0
AKT1	0	50
AKT2	16.67	0
BRAF	33.33	0
CDK4	50	0
CDK6	16.67	0
EGFR	16.67	0
ERBB2	0	0
FGFR1	33.33	50
FGFR2	0	0
FGFR3	0	0
FLT3	0	0
ITK	16.67	50
KDR	16.67	0
KIT	16.67	0
LCK	16.67	50
MET	16.67	0
NTRK1	16.67	0
PDGFRA	0	0
PDGFRB	0	0
PIM1	50	0
RAF1	33.33	0
RET	0	0
SYK	0	0

Supplementary Table S25. Implication of potential effects of Mg^{2+} ions on RNAi in liver cancer. We set "Select Cancer Study", "Select Genomic Profiles", "Enter a z-score threshold", and "Select Patient/Case Set" to "Liver Hepatocellular Carcinoma (TCGA, provisional)", "mRNA Expression z-Scores (RNA Seq V2 RSEM)", 1.0, and "Tumors with mRNA data (RNA Seq V2)", respectively, in cBioportal.org. According to high or low *TRPM7* expression, we divided the cancer patients (total 27 patients).

**25 oncogenic kinases**	***TRPM7* up-regulated patients (12 pts) : Mg^{2+} high patients (%)**	***TRPM7* down-regulated patients (15 pts) : Mg^{2+} low patients (%)**
ABL1	25	0
ABL2	50	13.33
AKT1	0	13.33
AKT2	50	13.33
BRAF	8.33	0
CDK4	8.33	33.33
CDK6	25	6.67
EGFR	25	0
ERBB2	16.67	13.33
FGFR1	0	0
FGFR2	16.67	6.67
FGFR3	16.67	0
FLT3	8.33	0
ITK	0	0
KDR	16.67	0
KIT	0	0
LCK	0	6.67
MET	16.67	13.33
NTRK1	0	6.67
PDGFRA	16.67	0
PDGFRB	16.67	0
PIM1	33.33	6.67
RAF1	16.67	6.67
RET	16.67	0
SYK	0	0

Supplementary Table S26. Implication of potential effects of Mg^{2+} ions on RNAi in lung adenocarcinoma. We set "Select Cancer Study", "Select Genomic Profiles", "Enter a z-score threshold", and "Select Patient/Case Set" to "Lung Adenocarcinoma (TCGA, Provisional)", "mRNA Expression z-Scores (RNA Seq V2 RSEM)", 1.0, and "Tumors with mRNA data (RNA Seq V2)", respectively, in cBioportal.org. According to high or low *TRPM7* expression, we divided the cancer patients (total 88 patients).

**25 oncogenic kinases**	***TRPM7* up-regulated patients (22 pts) : Mg^{2+} high patients (%)**	***TRPM7* down-regulated patients (66 pts) : Mg^{2+} low patients (%)**
ABL1	13.64	9.09
ABL2	13.64	19.70
AKT1	0.00	34.85
AKT2	9.09	24.24
BRAF	22.73	21.21
CDK4	22.73	34.85
CDK6	27.27	22.73
EGFR	36.36	21.21
ERBB2	31.82	24.24
FGFR1	9.09	12.12
FGFR2	27.27	3.03
FGFR3	27.27	13.64
FLT3	18.18	6.06
ITK	27.27	3.03
KDR	9.09	13.64
KIT	9.09	13.64
LCK	22.73	1.52
MET	18.18	34.85
NTRK1	9.09	3.03
PDGFRA	4.55	4.55
PDGFRB	0.00	12.12
PIM1	13.64	19.70
RAF1	18.18	10.61
RET	4.55	18.18
SYK	9.09	9.09

Supplementary Table S27. Implication of potential effects of Mg^{2+} ions on RNAi in lung squamous cell carcinoma (1). We set “Select Cancer Study”, “Select Genomic Profiles”, “Enter a z-score threshold”, and “Select Patient/Case Set” to “Lung Squamous Cell Carcinoma (TCGA, Nature 2012)”, “mRNA Expression z-Scores (RNA Seq V2 RPKM)”, 1.0, and “Tumors with mRNA data (RNA Seq)”, respectively, in cBioportal.org. According to high or low *TRPM7* expression, we divided the cancer patients (total 60 patients).

**25 oncogenic kinases**	***TRPM7* up-regulated patients (36 pts) : Mg^{2+} high patients (%)**	***TRPM7* down-regulated patients (24 pts) : Mg^{2+} low patients (%)**
ABL1	22.22	8.33
ABL2	25.00	8.33
AKT1	33.33	25.00
AKT2	27.78	29.17
BRAF	41.67	20.83
CDK4	19.44	16.67
CDK6	13.89	16.67
EGFR	36.11	16.67
ERBB2	2.78	4.17
FGFR1	11.11	8.33
FGFR2	30.56	0.00
FGFR3	11.11	12.50
FLT3	5.56	8.33
ITK	8.33	8.33
KDR	8.33	12.50
KIT	5.56	8.33
LCK	11.11	8.33
MET	11.11	4.17
NTRK1	11.11	12.50
PDGFRA	19.44	0.00
PDGFRB	5.56	0.00
PIM1	13.89	12.50
RAF1	5.56	20.83
RET	0.00	12.50
SYK	19.44	8.33

Supplementary Table S28. Implication of potential effects of Mg^{2+} ions in lung squamous cell carcinoma (2). We set "Select Cancer Study", "Select Genomic Profiles", "Enter a z-score threshold", and "Select Patient/Case Set" to "Lung Squamous Cell Carcinoma (TCGA, Provisional)", "mRNA Expression z-Scores (RNA Seq V2 RSEM)", 1.0, and "Tumors with mRNA data (RNA Seq V2 RSEM)", respectively, in cBioportal.org. According to high or low *TRPM7* expression, we divided the cancer patients (total 118 patients).

**25 oncogenic kinases**	***TRPM7*** **up-regulated patients (74 pts) : Mg^{2+} high patients (%)**	***TRPM7*** **down-regulated patients (44 pts) : Mg^{2+} low patients (%)**
ABL1	31.08	13.64
ABL2	25.68	13.64
AKT1	21.62	27.27
AKT2	21.62	36.36
BRAF	37.84	20.45
CDK4	12.16	18.18
CDK6	18.92	18.18
EGFR	37.84	20.45
ERBB2	4.05	9.09
FGFR1	24.32	11.36
FGFR2	24.32	2.27
FGFR3	16.22	15.91
FLT3	1.35	6.82
ITK	6.76	4.55
KDR	5.41	15.91
KIT	8.11	6.82
LCK	13.51	13.64
MET	4.05	11.36
NTRK1	6.76	6.82
PDGFRA	18.92	6.82
PDGFRB	4.05	4.55
PIM1	10.81	22.73
RAF1	6.76	18.18
RET	0.00	11.36
SYK	17.57	6.82

Supplementary Table S29. Implication of potential effects of Mg^{2+} ions on RNAi in ovarian serous cystadenocarcinoma (1). We set "Select Cancer Study", "Select Genomic Profiles", "Enter a z-score threshold", and "Select Patient/Case Set" to "Ovarian Serous Cystadenocarcinoma (TCGA, Nature 2011)", "mRNA Expression Z-scores (all genes)", 1.0, and "Tumors with mRNA data (Agilent microarray)", respectively, in cBioportal.org. According to high or low *TRPM7* expression, we divided the cancer patients (total 267 patients).

**25 oncogenic kinases**	***TRPM7* up-regulated patients (50 pts) : Mg^{2+} high patients (%)**	***TRPM7* down-regulated patients (217 pts) : Mg^{2+} low patients (%)**
ABL1	2.00	11.52
ABL2	12.00	18.89
AKT1	18.00	19.82
AKT2	28.00	28.11
BRAF	30.00	26.27
CDK4	28.00	23.50
CDK6	20.00	21.20
EGFR	20.00	15.67
ERBB2	6.00	11.98
FGFR1	10.00	20.74
FGFR2	8.00	12.90
FGFR3	8.00	24.42
FLT3	12.00	14.75
ITK	16.00	10.60
KDR	6.00	10.14
KIT	6.00	17.05
LCK	14.00	13.36
MET	20.00	17.97
NTRK1	8.00	28.11
PDGFRA	8.00	12.90
PDGFRB	10.00	12.44
PIM1	20.00	21.66
RAF1	22.00	15.21
RET	10.00	10.14
SYK	10.00	12.44

Supplementary Table S30. Implication of potential effects of Mg^{2+} ions on RNAi in ovarian serous cystadenocarcinoma (2). We set "Select Cancer Study", "Select Genomic Profiles", "Enter a z-score threshold", and "Select Patient/Case Set" to "Ovarian Serous Cystadenocarcinoma (TCGA, Provisional)", "mRNA Expression z-Scores (RNA Seq V2 RSEM)", 1.0, and "Tumors with mRNA data (RNA Seq V2 RSEM)", respectively, in cBioportal.org. According to high or low *TRPM7* expression, we divided the cancer patients (total 126 patients).

**25 oncogenic kinases**	***TRPM7* up-regulated patients (24 pts) : Mg^{2+} high patients (%)**	***TRPM7* down-regulated patients (102 pts) : Mg^{2+} low patients (%)**
ABL1	4.17	10.78
ABL2	33.33	24.51
AKT1	33.33	18.63
AKT2	25.00	26.47
BRAF	16.67	25.49
CDK4	4.17	18.63
CDK6	20.83	21.57
EGFR	25.00	9.80
ERBB2	4.17	13.73
FGFR1	12.50	17.65
FGFR2	25.00	9.80
FGFR3	4.17	5.88
FLT3	16.67	13.73
ITK	20.83	7.84
KDR	4.17	12.75
KIT	4.17	11.76
LCK	8.33	9.80
MET	20.83	16.67
NTRK1	8.33	4.90
PDGFRA	0.00	3.92
PDGFRB	4.17	11.76
PIM1	12.50	18.63
RAF1	41.67	17.65
RET	0.00	0.98
SYK	16.67	11.76

Supplementary Table S31. Implication of potential effects of Mg^{2+} ions on RNAi in pancreatic adenocarcinoma. We set "Select Cancer Study", "Select Genomic Profiles", "Enter a z-score threshold", and "Select Patient/Case Set" to "Pancreatic Adenocarcinoma (TCGA, Provisional)", "mRNA Expression z-Scores (RNA Seq V2 RSEM)", 1.0, and "Tumors with mRNA data (RNA Seq V2 RSEM)", respectively, in cBioportal.org. According to high or low *TRPM7* expression, we divided the cancer patients (total 14 patients).

**25 oncogenic kinases**	***TRPM7* up-regulated patients (7 pts) : Mg^{2+} high patients (%)**	***TRPM7* down-regulated patients (7 pts) : Mg^{2+} low patients (%)**
ABL1	28.57	0.00
ABL2	42.86	0.00
AKT1	14.29	14.29
AKT2	28.57	14.29
BRAF	28.57	14.29
CDK4	14.29	42.86
CDK6	28.57	14.29
EGFR	0.00	28.57
ERBB2	0.00	28.57
FGFR1	14.29	0.00
FGFR2	0.00	0.00
FGFR3	14.29	0.00
FLT3	14.29	14.29
ITK	28.57	0.00
KDR	28.57	0.00
KIT	14.29	14.29
LCK	14.29	0.00
MET	0.00	42.86
NTRK1	28.57	14.29
PDGFRA	14.29	0.00
PDGFRB	14.29	0.00
PIM1	14.29	14.29
RAF1	0.00	14.29
RET	42.86	0.00
SYK	14.29	28.57

Supplementary Table S32. Implication of potential effects of Mg^{2+} ions on RNAi in prostate adenocarcinoma (1). We set "Select Cancer Study", "Select Genomic Profiles", "Enter a z-score threshold", and "Select Patient/Case Set" to "Prostate Adenocarcinoma (MSKCC, Cancer Cell 2010)", "mRNA Expression Z-Scores vs Normals", 1.0, and "All Complete Tumors", respectively, in cBioportal.org. According to high or low *TRPM7* expression, we divided the cancer patients (total 32 patients).

**25 oncogenic kinases**	***TRPM7*** **up-regulated patients (14 pts) : Mg^{2+} high patients (%)**	***TRPM7*** **down-regulated patients (18 pts) : Mg^{2+} low patients (%)**
ABL1	14.29	0.00
ABL2	78.57	22.22
AKT1	7.14	16.67
AKT2	14.29	11.11
BRAF	92.86	0.00
CDK4	21.43	22.22
CDK6	35.71	16.67
EGFR	14.29	5.56
ERBB2	14.29	5.56
FGFR1	7.14	0.00
FGFR2	0.00	0.00
FGFR3	14.29	66.67
FLT3	14.29	77.78
ITK	42.86	72.22
KDR	14.29	5.56
KIT	14.29	0.00
LCK	0.00	72.22
MET	14.29	5.56
NTRK1	0.00	72.22
PDGFRA	14.29	0.00
PDGFRB	7.14	5.56
PIM1	28.57	11.11
RAF1	50.00	0.00
RET	21.43	55.56
SYK	14.29	22.22

Supplementary Table S33. Implication of potential effects of Mg^{2+} ions on RNAi in prostate adenocarcinoma (2). We set "Select Cancer Study", "Select Genomic Profiles", "Enter a z-score threshold", and "Select Patient/Case Set" to "Prostate Adenocarcinoma (TCGA, Provisional)", "mRNA Expression z-Scores (RNA Seq V2 RSEM)", 1.0, and "Tumors with mRNA data (RNA Seq V2)", respectively, in cBioportal.org. According to high or low *TRPM7* expression, we divided the cancer patients (total 63 patients).

**25 oncogenic kinases**	***TRPM7* up-regulated patients (29 pts) : Mg^{2+} high patients (%)**	***TRPM7* down-regulated patients (34 pts) : Mg^{2+} low patients (%)**
ABL1	10.34	23.53
ABL2	51.72	8.82
AKT1	3.45	26.47
AKT2	6.90	23.53
BRAF	65.52	2.94
CDK4	3.45	32.35
CDK6	27.59	2.94
EGFR	48.28	2.94
ERBB2	0.00	11.76
FGFR1	0.00	32.35
FGFR2	10.34	14.71
FGFR3	6.90	8.82
FLT3	17.24	2.94
ITK	6.90	2.94
KDR	20.69	5.88
KIT	20.69	11.76
LCK	3.45	0.00
MET	17.24	2.94
NTRK1	3.45	14.71
PDGFRA	41.38	11.76
PDGFRB	13.79	14.71
PIM1	10.34	14.71
RAF1	6.90	11.76
RET	10.34	5.88
SYK	13.79	11.76

Supplementary Table S34. Implication of potential effects of Mg^{2+} ions on RNAi in cutaneous melanoma. We set "Select Cancer Study", "Select Genomic Profiles", "Enter a z-score threshold", and "Select Patient/Case Set" to "Skin Cutaneous Melanoma (TCGA, Provisional)", "mRNA Expression z-Scores (RNA Seq V2 RSEM)", 1.0, and "Tumors with mRNA data (RNA Seq V2)", respectively, in cBioportal.org. According to high or low *TRPM7* expression, we divided the cancer patients (total 101 patients).

**25 oncogenic kinases**	***TRPM7* up-regulated patients (43 pts) : Mg^{2+} high patients (%)**	***TRPM7* down-regulated patients (58 pts) : Mg^{2+} low patients (%)**
ABL1	0.00	17.24
ABL2	55.81	20.69
AKT1	2.33	29.31
AKT2	2.33	32.76
BRAF	44.19	10.34
CDK4	6.98	43.10
CDK6	23.26	5.17
EGFR	4.65	13.79
ERBB2	16.28	27.59
FGFR1	4.65	6.90
FGFR2	0.00	1.72
FGFR3	4.65	17.24
FLT3	2.33	1.72
ITK	4.65	5.17
KDR	6.98	5.17
KIT	11.63	18.97
LCK	4.65	5.17
MET	13.95	12.07
NTRK1	4.65	22.41
PDGFRA	4.65	5.17
PDGFRB	6.98	15.52
PIM1	2.33	15.52
RAF1	27.91	18.97
RET	6.98	12.07
SYK	2.33	5.17

Supplementary Table S35. Implication of the potential effects of Mg^{2+} ions on RNAi in stomach cancer. We set "Select Cancer Study", "Select Genomic Profiles", "Enter a z-score threshold", and "Select Patient/Case Set" to "Stomach Adenocarcinoma (TCGA, Provisional)", "mRNA expression z-Scores (RNA Seq V2 RSEM)", 1.0, and "Tumors with mRNA data (RNA Seq V2)", respectively, in cBioportal.org. According to high or low *TRPM7* expression, we divided the cancer patients (total 91 patients).

**25 oncogenic kinases**	***TRPM7* up-regulated patients (33 pts) : Mg^{2+} high patients (%)**	***TRPM7* down-regulated patients (58 pts) : Mg^{2+} low patients (%)**
ABL1	6.06	8.62
ABL2	21.21	8.62
AKT1	3.03	24.14
AKT2	6.06	43.10
BRAF	45.45	12.07
CDK4	12.12	39.66
CDK6	45.45	8.62
EGFR	33.33	10.34
ERBB2	33.33	31.03
FGFR1	6.06	13.79
FGFR2	30.30	12.07
FGFR3	9.09	15.52
FLT3	15.15	3.45
ITK	12.12	1.72
KDR	12.12	12.07
KIT	12.12	3.45
LCK	6.06	12.07
MET	27.27	8.62
NTRK1	6.06	17.24
PDGFRA	24.24	1.72
PDGFRB	6.06	17.24
PIM1	6.06	15.52
RAF1	39.39	12.07
RET	12.12	5.17
SYK	21.21	3.45

Supplementary Table S36. Implication of potential effects of Mg^{2+} ions on RNAi in thyroid cancer. We set “Select Cancer Study”, “Select Genomic Profiles”, “Enter a z-score threshold”, and “Select Patient/Case Set” to “Thyroid Carcinoma (TCGA, Provisional)”, “mRNA expression z-Scores (RNA Seq V2 RSEM)”, 1.0, and “Tumors with mRNA data (RNA Seq V2)”, respectively, in cBioportal.org. According to high or low *TRPM7* expression, we divided the cancer patients (total 138 patients).

**25 oncogenic kinases**	***TRPM7* up-regulated patients (69 pts) : Mg^{2+} high patients (%)**	***TRPM7* down-regulated patients (69 pts) : Mg^{2+} low patients (%)**
ABL1	21.74	10.14
ABL2	7.25	5.80
AKT1	7.25	23.19
AKT2	26.09	10.14
BRAF	31.88	4.35
CDK4	11.59	43.48
CDK6	20.29	2.90
EGFR	18.84	4.35
ERBB2	0.00	26.09
FGFR1	26.09	7.25
FGFR2	21.74	4.35
FGFR3	1.45	21.74
FLT3	5.80	0.00
ITK	7.25	0.00
KDR	23.19	13.04
KIT	23.19	5.80
LCK	7.25	4.35
MET	4.35	7.25
NTRK1	0.00	1.45
PDGFRA	7.25	4.35
PDGFRB	11.59	10.14
PIM1	13.04	14.49
RAF1	23.19	4.35
RET	0.00	10.14
SYK	24.64	1.45

Supplementary Table S37. Implication of the potential effects of Mg^{2+} ions on RNAi in endometrial cancer. We set "Select Cancer Study", "Select Genomic Profiles", "Enter a z-score threshold", and "Select Patient/Case Set" to "Uterine Corpus Endometrial Carcinoma (TCGA, Provisional)", "mRNA expression z-Scores (RNA Seq V2 RSEM)", 1.0, and "Tumors with mRNA data (RNA Seq V2)", respectively, in cBioportal.org. According to high or low *TRPM7* expression, we divided the cancer patients (total 123 patients).

**25 oncogenic kinases**	***TRPM7* up-regulated patients (54 pts) : Mg^{2+} high patients (%)**	***TRPM7* down-regulated patients (69 pts) : Mg^{2+} low patients (%)**
ABL1	16.67	11.59
ABL2	46.30	23.19
AKT1	7.41	49.28
AKT2	7.41	57.97
BRAF	31.48	14.49
CDK4	11.11	23.19
CDK6	7.41	10.14
EGFR	33.33	11.59
ERBB2	7.41	31.88
FGFR1	20.37	31.88
FGFR2	12.96	10.14
FGFR3	1.85	13.04
FLT3	9.26	11.59
ITK	5.56	2.90
KDR	1.85	5.80
KIT	12.96	0.00
LCK	1.85	13.04
MET	18.52	7.25
NTRK1	5.56	8.70
PDGFRA	3.70	7.25
PDGFRB	3.70	18.84
PIM1	3.70	17.39
RAF1	24.07	20.29
RET	3.70	7.25
SYK	18.52	7.25

References

1. Wang Y, Juranek S, Li H, Sheng G, Wardle GS, et al. (2009) Nucleation, propagation and cleavage of target RNAs in Ago silencing complexes. Nature 461: 754-761.
2. Schirle NT, MacRae IJ (2012) The crystal structure of human Argonaute2. Science 336: 1037-1040.
